# The virome of German bats: comparing virus discovery approaches

**DOI:** 10.1038/s41598-021-86435-4

**Published:** 2021-04-01

**Authors:** Claudia Kohl, Annika Brinkmann, Aleksandar Radonić, Piotr Wojtek Dabrowski, Kristin Mühldorfer, Andreas Nitsche, Gudrun Wibbelt, Andreas Kurth

**Affiliations:** 1 Centre for Biological Threats and Special Pathogens, Robert Koch Institute, Seestrasse 10, 13353 Berlin, Germany; 2 Robert Koch Institute, Methodology and Research Infrastructure 2, Genome Sequencing, Berlin, Germany; 3 Robert Koch Institute, Methodology and Research Infrastructure 1, Bioinformatics, Berlin, Germany; 4 Leibniz Institute for Zoo and Wildlife Research, Berlin, Germany

**Keywords:** Viral genetics, Viral reservoirs

## Abstract

Bats are known to be reservoirs of several highly pathogenic viruses. Hence, the interest in bat virus discovery has been increasing rapidly over the last decade. So far, most studies have focused on a single type of virus detection method, either PCR, virus isolation or virome sequencing. Here we present a comprehensive approach in virus discovery, using all three discovery methods on samples from the same bats. By family-specific PCR screening we found sequences of paramyxoviruses, adenoviruses, herpesviruses and one coronavirus. By cell culture we isolated a novel bat adenovirus and bat orthoreovirus. Virome sequencing revealed viral sequences of ten different virus families and orders: three bat nairoviruses, three phenuiviruses, one orbivirus, one rotavirus, one orthoreovirus, one mononegavirus, five parvoviruses, seven picornaviruses, three retroviruses, one totivirus and two thymoviruses were discovered. Of all viruses identified by family-specific PCR in the original samples, none was found by metagenomic sequencing. Vice versa, none of the viruses found by the metagenomic virome approach was detected by family-specific PCRs targeting the same family. The discrepancy of detected viruses by different detection approaches suggests that a combined approach using different detection methods is necessary for virus discovery studies.

## Introduction

Bats have been recognized as potential reservoir host of several highly pathogenic viruses like Hendra virus, Nipah virus, Marburg virus and SARS-CoV viruses^1–5^. With more than 60 million years of evolution they belong to the oldest mammals we know today^6^. Furthermore, bats are often discussed as being special in the way they react to infections. Bat immunology is aiming to shed light on the very special way bats and pathogens coevolve^7–9^. Numerous publications and studies are focusing on virus discovery in this very distinct mammalian order and accelerate the publication of thousands of novel viral sequences. While mainly family-specific PCR approaches were used to detect novel virus strains during the first years of virus discovery in bats, next generation sequencing approaches have gained in significance in recent years. The metagenomic view on viruses (virome) supports the simultaneous detection of viruses in individual or pooled samples within a very short time. However, for samples with a high host nucleotide background it became apparent that some sort of purification procedure might be necessary for NGS approaches to increase significantly the number of obtained viral sequences from biological samples. Various protocols have been published to address this problem^10–12^.

By now, already 16 bat virome studies are available at PubMed and numerous additional ones are presently being conducted. In most studies guano, feces or oral swabs were used as sample sources^13–25^ (Table 1). Bat organ tissues were used for only three studies which were performed in Southeast China, Myanmar^26,27^ and France^28^. In the first two studies, either bought or trapped live bats of different Asian species were used after immediate dissecting^26^. The latter study was carried out in France where only nine bat carcasses belonging to five species were used^28^. Presently, the database of bat-associated viruses lists more than 800 viruses in European bats, mostly detected by family-specific PCRs^29^.

**Table 1:** Comparison of results from virus discovery studies conducted.

Origin	Europe		Asia		NZ	Americas	Asia							Africa			
Sample type	Carcasses		Organs		Guano	Feces and saliva											
Reference	E	(28)	(26)	(27)	(16)	(18)	(21)	(22)	(20)	(19)	(15)	(24)	(14)	(23)	(13)	(17)	(25)
Adenovirus	A		C	C		C				C							
Astrovirus			C	C		C				C							
Bunyavirus	B	C								C							
Bocavirus								C									
Calicivirus				C	C					C							C
Circovirus				C		C	C	C		C							
Coronavirus	A			C		C			C	C	C	C					
Flavivirus		C	C	C				C		C							
Hepadnavirus										C							
Hepevirus				C	C					C							
Herpesvirus	A	C	C	C				C		C					C		
Nairovirus	B	C					C										
Nodavirus						C											
Orbivirus	B																
Orthomyxovirus										C							
Orthoreovirus	B											C					
Papillomavirus				C	C			C		C		C		C	C	C	
Paramyxovirus	A									C							
Parvovirus	B		C	C		C				C	C						
Phlebovirus	B																
Picornavirus	B		C	C		C		C		C							
Polyomavirus					C					C			C				
Poxvirus		C	C	C		C									C		
Reovirus	B	C		C						C		C					
Retrovirus	B	C		C			C					C	C				
Retrovirus			C					C		C							
Rhabdovirus										C							
Rotavirus	B			C								C					
Tymovirus	B								C								
Togavirus				C													

The viral sequences obtained by family-specific PCR are marked with “A” and viral sequences obtained by virome sequencing are marked with “B”. Adenovirus and reoviruses were also isolated. The “C” indicates the finding of the respective viruses found by other studies.

*E* the results from this study, *NZ* New Zealand.

This paper summarizes comprehensive results obtained during a study on German bats by three different detection approaches, namely PCR, cell culture virus isolation and NGS. The results of virome sequencing of 16 different bat species are highlighted. Furthermore, we discuss the suggestion of the virome sequencing approach replacing conventional virus discovery methods such as family-specific PCR and cell culture virus isolation.

## Results

### Screening with family-specific PCRs

The PCR screening resulted in the detection of several novel viral sequences for paramyxoviruses, adenoviruses, herpesviruses and coronavirus (Table 2). Sequences found for paramyxoviruses, adenoviruses (polymerase) and herpesviruses have been published before^30–34^. A novel coronavirus (CoV) sequence is described here for the first time. No arenaviruses, filoviruses, nairo- and phenuiviruses, hantaviruses and flaviviruses were detected by PCR.

**Table 2:** Results PCR screening. Bats were screened for the presence of virus nucleic acids by different PCR assays.

PCR assays	Assay type	Bats #/novel/size*	Assay reference	Virus reference
***Adenoviridae***				
Adenoviridae (Polymerase)	Pan	79/14/203^H^	(67)	(31)
***Arenaviridae***				
Old-World	Pan	0/0/60	(61)	Unpublished
***Bunyaviridae***				
Hanta Virus Puumala	cPCR	0/0/150	In-house design RKI	(71)
Hanta Virus Dobrava	cPCR	0/0/150	In-house design RKI	(71)
Hanta Virus Tula	cPCR	0/0/150	In-house design RKI	(71)
Hantaviruses	Pan	0/0/180	(64)	Unpublished
Nairoviruses	Pan	0/0/150	(65)	Unpublished
Phleboviruses	Pan	0/0/150	(65)	Unpublished
***Coronaviridae***				
Coronaviridae	Pan	1/1/240	(35)	Unpublished (MN851285)
Coronaviridae	Pan	0/0/90	In-house design RKI	unpublished
***Flaviviridae***				
Flaviviridae	Pan	0/0/150	In-house design	Unpublished
Flaviviridae	Pan	0/0/180	(63)	(71)
***Herpesviridae***				
BatGHV1	cPCR	1/1/180	(34)	(71)
BatGHV3	cPCR	7/1/180	(34)	(71)
BatGHV4	cPCR	22/1/210	(34)	(71)
BatGHV5	cPCR	11/1/210	(34)	(71)
BatGHV6	cPCR	24/1/210	(34)	(71)
BatGHV7	cPCR	2/1/210	(34)	(71)
BatBHV1	cPCR	1/1/180	(34)	(71)
Herpesviridae	pan	11/9/60	(68)	Unpublished
***Orthomyxoviridae***				
Influenza A	qPCR	0/0/330	(66)	–
***Paramyxoviridae***				
Res-Mor-Hen	Pan	2/2/120	(60)	(32)
PAR	Pan	3/3/180	(60)	(32)
***Poxviridae***				
Low GC poxviruses	Pan	0/0/263	(70)	Unpublished

*pan* generic family-specific assay, *qPCR* quantitative real-time PCR, *cPCR* conventional specific PCR. *#/novel/size: #, number of positive samples; novel, number of novel viruses obtained; size, bat sample size. ^H^Screened in collaboration with our Hungarian colleagues (see Ref.^33^).

#### Coronavirus

A novel coronavirus sequence was obtained using the family-specific CoV PCR published earlier by de Souza Luna et al*.*^35^. Bat number E210/09 (*Pipistrellus pipistrellus*) was found to be positive for CoV in the intestinal sample. Phylogenetic analysis revealed that the sequence was most closely related to alphacoronaviruses of group 1 (Supplementary Fig. S1). The sequence (296 nt) was named Bat CoV 210/09 P.pip and is available under accession number MN851285.

### Virus isolation in cell culture

Virus isolation was attempted from all 375 samples in either two out of six different cell culture systems. Two novel isolates were obtained, Bat orthoreovirus T3/Bat/Germany/342/08 and Bat adenovirus 2^30,31,36^. Both viruses had initially been cultivated in Vero cell lines (Supplementary Table S1).

### Virome sequencing and phylogenetic reconstruction

**Figure 1:**
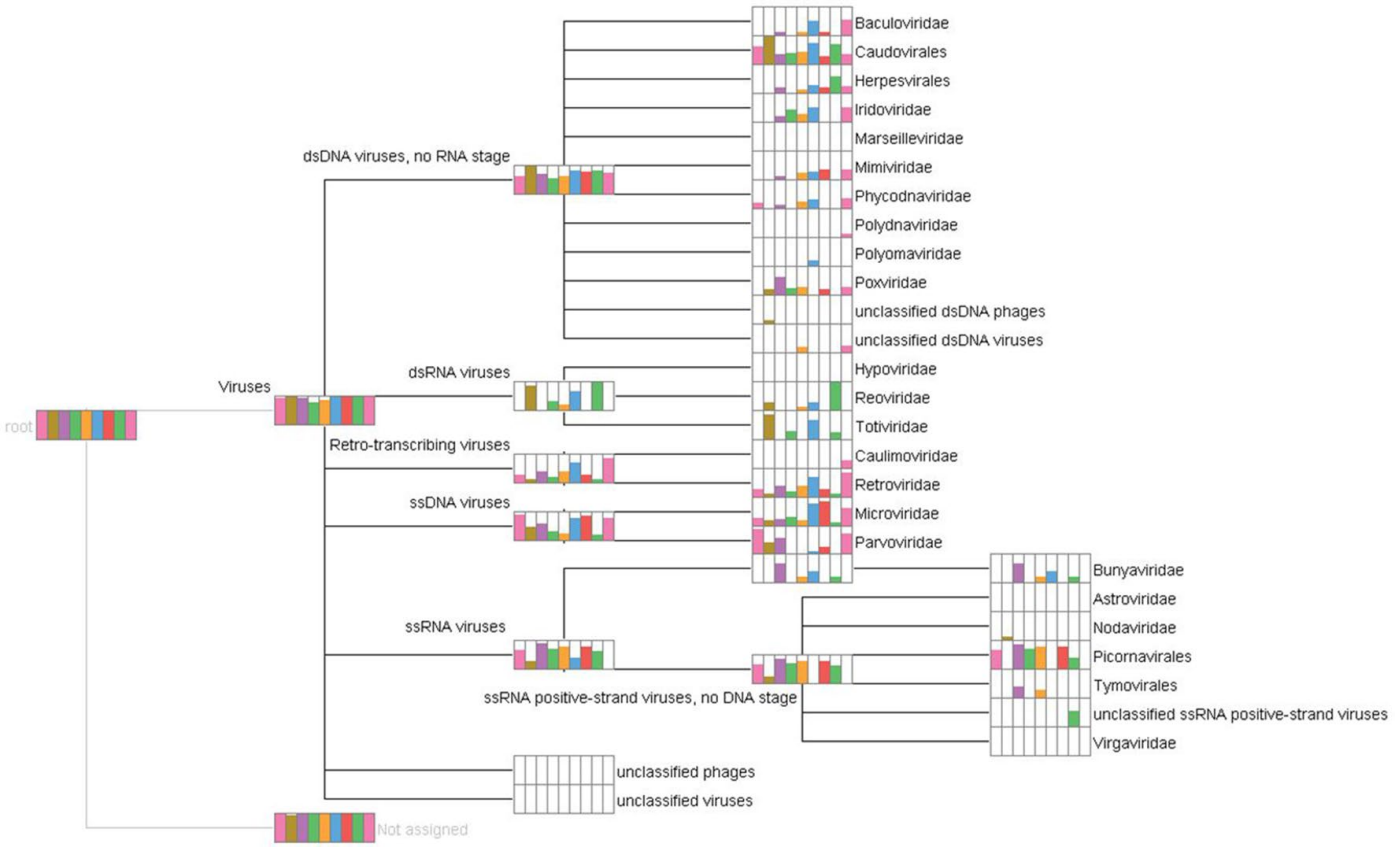
Comparison of all pools with MEGAN6. The different colors stand for the individual pools: pool 1 pink, pool 2 light brown, pool 3 brown, pool 4 light green, pool 5 yellow, pool 6 blue, pool 7 red, pool 8 green, pool 9 light pink. The height of the color bars indicates the percentage of viruses found in the corresponding nodes of the taxonomic tree.

**Figure 2:**
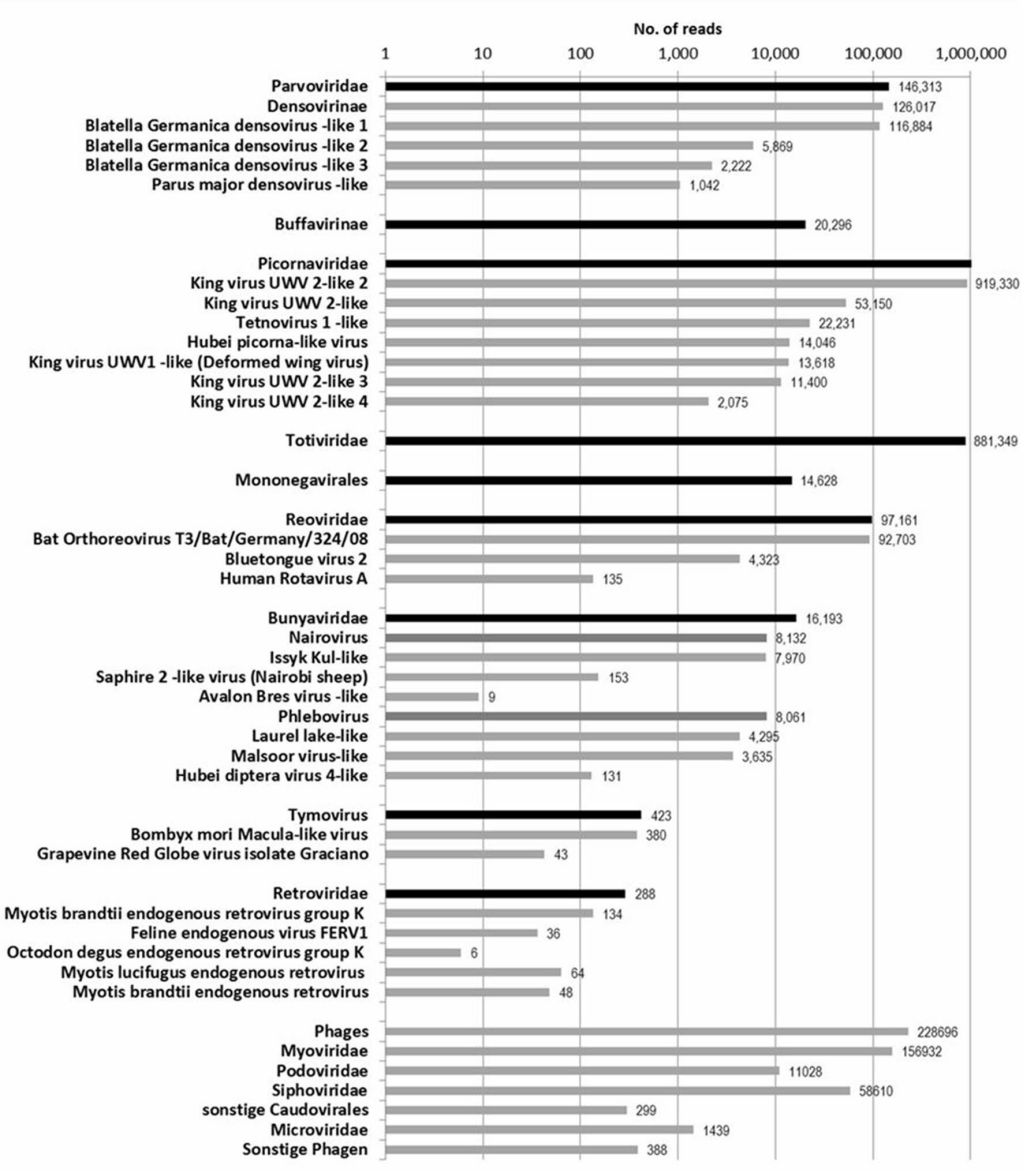
Schematic summary of the number of reads related to viruses classified by viral genus, subfamily and family. Viruses that did not meet the quality criteria were excluded.

Sequencing of the nine bat pools via Illumina HiSeq revealed 127,338,644 reads after trimming. In Fig. 1 the raw output obtained from MEGAN6^37^ is depicted. Though MEGAN6 displayed a large number of hits for different viral families, several hits did not meet the quality criteria. These reads could not be remapped to reference sequences, revealed non-plausible BLASTx/BLASTn results (i.e. genomic DNA of bats) or shared 100 percent identity to sequences obtained before (possible cross-contamination). Supplementary Table S2 summarizes the number of filtered reads (length/quality) obtained per pool with the number of viral reads (w/o phages) finally checked for quality and allocated by Diamond^38^, MEGAN6 and further analysis. In Fig. 2 the overall number of viral reads in all pools per virus family is summarized.

Viruses of nine different families and orders (*Parvoviridae*, *Picornaviridae*, *Totiviridae*, *Mononegavirales*, *Reoviridae*, *Bunyavirales*, *Tymovirus*, *Retroviridae* and phages) were confirmed within the bat samples by virome sequencing and further analysis. For each family, selected viruses were retested by specific PCRs in the individual bats’ organs. For the viruses of highest interest, phylogenetic reconstructions were calculated as described in the methods section.

All viruses were confirmed by sequence assembly and comparison to reference strains and to each other as described in the methods section. Most of the viruses were tested back with designed specific primers, PCR and Sanger sequencing. The individual length of obtained contigs, the accession number of the reference sequence and the pairwise identity on nt and aa level are summarized in Table 3. The accession numbers of all obtained viral sequences are available in Table 4. The full description of the results is given in the supplemental section.

**Table 3:** Virus results per virus family.

	Virus/Acc No of best match	#	Species	Reads	Longest contig	ID nt/aa %
*Parvoviridae*	Blatella Germanica densovirus-like 1	1	*Myotis myotis*	116,884	3727 nt	97.0/98.0
	JQ320376.1		*Pipistrellus kuhlii*		98.5% genome	
			*Myotis nattereri*			
	Blatella Germanica densovirus-like 2	3	*Eptesicus nilssonii*	5869	614 nt	98.0/99.0
	KU727766.1					
	Blatella Germanica densovirus-like 3	9	*Myotis daubentonii*	2222	983 nt	99.0/99.0
	JQ320376.1		*Vespertilio murinus*			
	Parus major densovirus-like	3	*Eptesicus nilssonii*	1042	1,568 nt	98.0/95.0
	KU727766				67.6% genome	
	Bat Bufavirus	2	*Nyctalus noctula*	20,296	722 nt	88.0/92.0
	Bat parvovirus isolate BtNv-PV				40.5% genome	
	KJ641683					
*Picornaviridae*	King virus UWV1-like	1	*Myotis myotis*	13,618	2355 nt	99.0/99.0
	KX779453		*Pipistrellus kuhlii*		40.3% genome	
			*Myotis nattereri*			
	King virus UWV 2-like	3	*Eptesicus nilssonii*	53,150	6010 nt	97.0/99.0
	KX779454.1			1022	100% genome	
	King virus UWV 2-like 2	5	*Pipistrellus pipistrellus*	919,330	2,438 nt	98.0/99.0
	KX779453.1					
	King virus UWV 2-like 3	8	*Plecotus auritus*	11,400	971 nt	97.0/98.0
	KX779453.1		*Pipistrellus nathusii*			
	Tetnovirus 1-like	2	*Nyctalus noctula*	22,231	710 nt	70.0/78.0
	HM480375	4	*Myotis mystacinus*	2507	46.7% genome	
	Hubei picorna-like virus	8	*Plecotus auritus*	14,046	481 nt	66.0 /59.0
	KX883698.1		*Pipistrellus nathusii*		4.01% genome	
	King virus UWV 2-like 4	7	*Eptesicus serotinus*	2075	1523 nt	98.80%
*Totiviridae*	Eimeria Tenella RNA virus 1-like	2	*Nyctalus noctula*	881,349	2059 nt	–/50.0
	KJ363185				81.3% genome	
Mononegavirus	Wenzhou tapeworm virus 1-like	2	*Nyctalus noctula*	14,628	627 nt	–/35.0
	KX884436				4% genome	
Reovirus	Irlbach Bat Orbivirus	2	*Nyctalus noctula*	4323	783 nt	70.69/81.0
	MH144554.1 (Bat Orbivirus China)				4% genome	
	Hannover bat rotavirus	5	*Pipistrellus pipistrellus*	135	424 nt	58.0/57.0
	GQ398017 (Human Rotavirus A)					
	Bat Orthoreovirus T3/Bat/Germany/324/08	8	*Pipistrellus nathusii*	92,703	1296 nt	100/100
	JQ412758		*Plecotus auritus*		90% genome	
Bunyavirus	Issyk-Kul virus strain PbGER	3	*Eptesicus nilssonii*	5,580	1281 nt	95.0/99.0
	KR709221.1 Issyk-Kul virus				93.5% segment	
	Zwiesel bat banyangvirus	3	*Eptesicus nilssonii*	3,635	2,578 nt	70.0/72.0
	KF186495.1 Malsoor virus, SFTS				90.0% genome	
	Berlin bat nairovirus	5	*Pipistrellus pipistrellus*	153	384 nt	69/58
	KU925485 Sapphire II-like virus					
	Bavarian bat lalavirus	8	*Pipistrellus nathusii*	131	176 nt	–/50.0
	NC-043671 Laurel Lake virus-like 1		*Plecotus auritus*			
	Wittenau bat nairovirus	6	*Pipistrellus pipistrellus*	9	377 nt	72 / 50
	KU925443 Avalon Bres virus		*Pipistrellus pygmaeus*			
	Munich bat lalavirus	8	*Pipistrellus nathusii*	4295	528 nt	–/43.0
	NC_043679.1 Laurel Lake-like virus 2		*Plecotus auritus*			
Tymovirus	Bombyx mori latent virus	3	*Eptesicus nilssonii*	380	1179 nt	–/50.0
	NC038331					
	Grapevine Red Globe virus isolate	5	*Pipistrellus pipistrellus*	43	320 nt	76.0/78.0
	KX109927.1					
*Retroviridae*	Myotis myotis endogenous retrovirus	1	*Myotis myotis*	134	664 nt	
	XM_014547948 Myotis brandtii ERV		*Pipistrellus kuhlii*			97.0/93.0
			*Myotis nattereri*			
	Pipistrellus pipistrellus endogenous Retrovirus XP_014403434 FERV1-like	6	*Pipistrellus pipistrellus*	36	558 nt	97.0/93.0
	Myotis daubentonii endogenous retrovirus XR_001346663.2 Myotis lucifugus endogenous retrovirus-like	9	*Myotis daubentonii* *Vespertilio murinus*	64	279 nt	95.0/90.2

Acc No, Accession number; #, Pool number; ID nt/aa, Identity nucleic acid/amino acid; nt, nucleotides. –, no homology was found.

**Table 4:** Names and accession numbers of novel virus sequences.

Acc. No.	Final name of novel virus	Related virus
MN851277	Blatella Germanica densovirus-like virus 2	Blatella Germanica densovirus
MN851278	Blatella Germanica densovirus-like virus 3	Blatella Germanica densovirus
MN851279	Parus major densovirus-like virus	Parus major densovirus
MN851280	King virus UWV 2-like virus	King virus UWV 2
MN851281	King virus UWV 2-like 2 virus	King virus UWV 2
MN851282	King virus UWV 2-like 3 virus	King virus UWV 2
MN851283	King virus UWV 2-like 4 virus	King virus UWV 2
MN851284	Tetno virus 1-like virus	Tetno virus 1
MN851285	Bat coronavirus 210/09 P.pip	Bat alphacoronaviruses
MN851286	Hubei picorna-like virus	Hubei picorna virus
MN851287	Eimeria Tenella RNA virus 1-like virus	Eimeria Tenella RNA virus 1
MN851288	Berlin bat mononegavirus (BbmV)	Wenzhou tapeworm 1 virus
MN851289	Irlbach bat orbivirus (IboV)	Sathuvachari virus
MN851290	Hannover bat rotavirus (HbrV)	Rotaviruses type A
MN851291	Bat orthoreovirus T3/Bat/Germany/342/08	Bat orthoreovirus T3/Bat/Germany/342/09
MN851292	Berlin bat nairovirus (BbnV)	Sapphire II virus
MN851293	Bat bufavirus	Bat parvovirus BtNv-PV/SC2013
MN851294	King virus UWV 1-like virus	King virus UWV 1
MN851295	Blatella Germanica densovirus-like virus 1	Blatella Germanica densovirus
MN851296	Wittenau bat nairovirus (WbnV)	Avalon Bres virus
MN851297	Munich bat lalavirus (MblV)	Laurel Lake virus
MN851298	Bombyx mori latent-like virus	Bombyx mori latent virus
MN851299	Grapevine red globe-like virus	Grapevine red globe virus
MN851300	*Pipistrellus pipistrellus* endogenous retrovirus	FERV1
MN851301	Issyk-Kul virus strain PbGER	Issyk-Kul virus
MN851301	*Myotis myotis* endogenous retrovirus	*Myotis brandtii* endogenous retrovirus
MN851302	*Myotis daubentonii* endogenous retrovirus	*Myotis lucifugus* endogenous retrovirus
MN851303	Zwiesel bat banyangvirus	Malsoor virus/SFTS

#### Parvoviridae

Parvoviruses (n = 5) were found in pools 1, 2, 3 and 9 belonging to the subspecies *Densovirinae* (146,313 reads) and *Parvovirinae* (20,296 reads). The virus sequences were named after the order of appearance and in relation to reference strains: Blatella Germanica densovirus-like virus 1, Blatella Germanica densovirus-like virus 2, Blatella Germanica densovirus-like virus 3, Parus major densovirus-like virus and Bat bufavirus (accession numbers: MN851295, MN851277, MN851278, MN851279 and MN851293).

#### Picornaviridae

Picornaviruses (n = 7) were found in pools 1, 2, 3, 4, 5, 7 and 8 (1,039,379 reads), all of which were related to King virus UWV1, King virus UWV2, Tetnovirus 1 or Hubei Picorna-like virus. The virus sequences were named after the order of appearance and in relation to reference strains: King virus UWV1-like virus, King virus UWV2-like virus 1, King virus UWV2-like virus 2, King virus UWV2-like virus 3, King virus UWV2-like virus 4, Tetnovirus 1-like virus and Hubei Picorna-like virus. Obtained sequences are available under the following accession numbers: MN851294, MN851280, MN851281, MN851282, MN851283, MN851284 and MN851286.

#### Totiviridae

One totivirus was found in pool 2 (881,349 reads) which appears to be most closely related to Eimeria tenella RNA virus 1. The virus sequence was named in relation to the reference strain: Picorna Eimeria tenella virus 1-like virus. The obtained sequence is available under the following accession number: MN851287.

#### Mononegavirus

A distinct sequence, related to mononegaviruses Wenzhou tapeworm virus and Midway virus, was identified in pool 2 (14,628 reads). The obtained sequence is available under the following accession number: MN851288. Phylogenetic reconstruction of 665 nt (polymerase) Wenzhou tapeworm virus-like virus in comparison to the reference strains of *Mononegavirales* is displayed in (Supplementary Fig. S2).

#### Reoviridae

**Figure 3:**
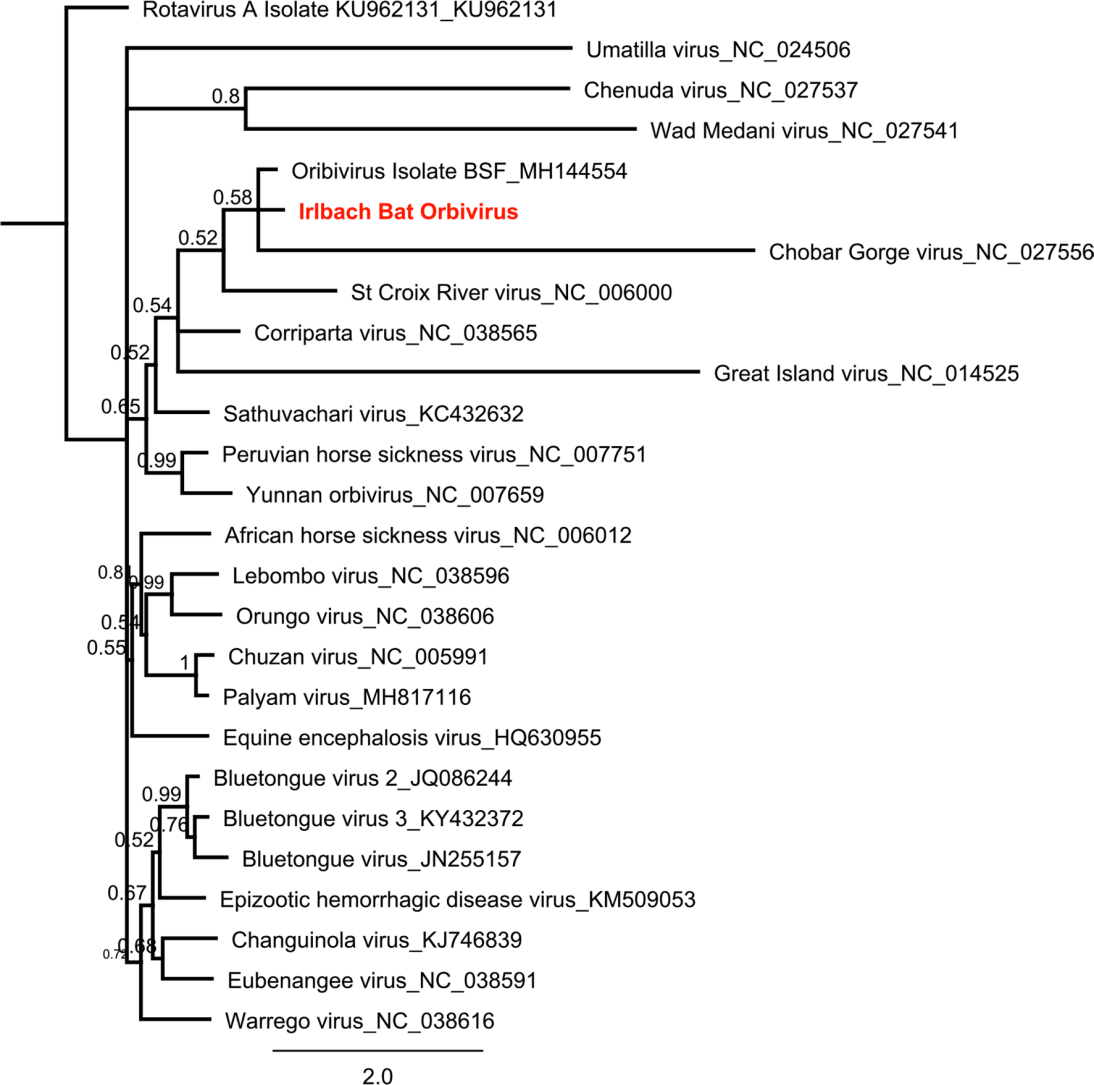
Phylogenetic reconstruction of Orbiviruses ML, 1 million, 780 nt of the VP4 protein. Reconstruction was performed via the Bayesian MCMC approach using MrBayes with the following settings (burn-in, 10%; frequency, 200; chain length, 1 million to 10 million, depending on when a standard derivation of below 0.025 was reached)^78^. Reconstructed trees were visualized using FigTree and posterior probabilities were depicted for each node (http://tree.bio.ed.ac.uk/software/figtree/).

**Figure 4:**
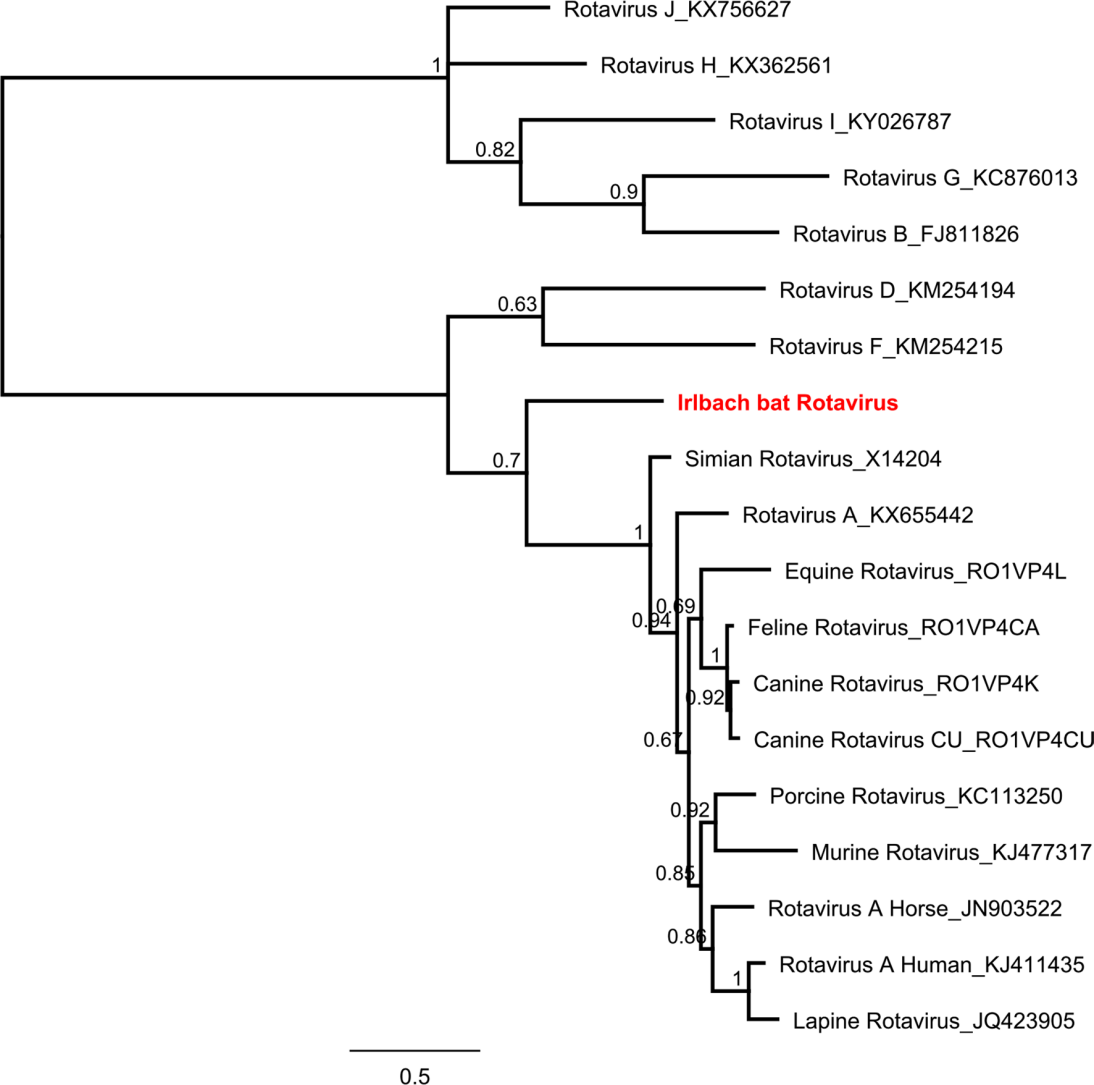
Phylogenetic reconstructions of Rotaviruses ML, 1 million, 450 nt of the VP4 protein. Reconstruction was performed via the Bayesian MCMC approach using MrBayes with the following settings (burn-in, 10%; frequency, 200; chain length, 1 million to 10 million, depending on when a standard derivation of below 0.025 was reached)^78^. Reconstructed trees were visualized using FigTree and posterior probabilities were depicted for each node (http://tree.bio.ed.ac.uk/software/figtree/).

Reoviruses (n = 3) were found in pools 2, 5 and 8 (97,161 reads) and include related reoviruses of three distinct genera orbivirus (related to Bat orbivirus China—unpublished AccNo. MH144554.1), rotavirus (related to Human rotavirus A-like virus) and orthoreovirus (Bat orthoreovirus T3/Bat/Germany/342/08). Bat orthoreovirus T3/Bat/Germany/342/08 had been isolated before from the same sample set; no further analysis was conducted here^30^. Obtained sequences are available under the following accession numbers: MN851289, MN851290 and MN851291. Phylogenetic reconstruction of orbiviruses (780 nt) and rotaviruses (450 nt, VP4) in comparison to the two novel sequences is displayed in Figs. 3 and 4, respectively.

#### Nairoviridae

**Figure 5:**
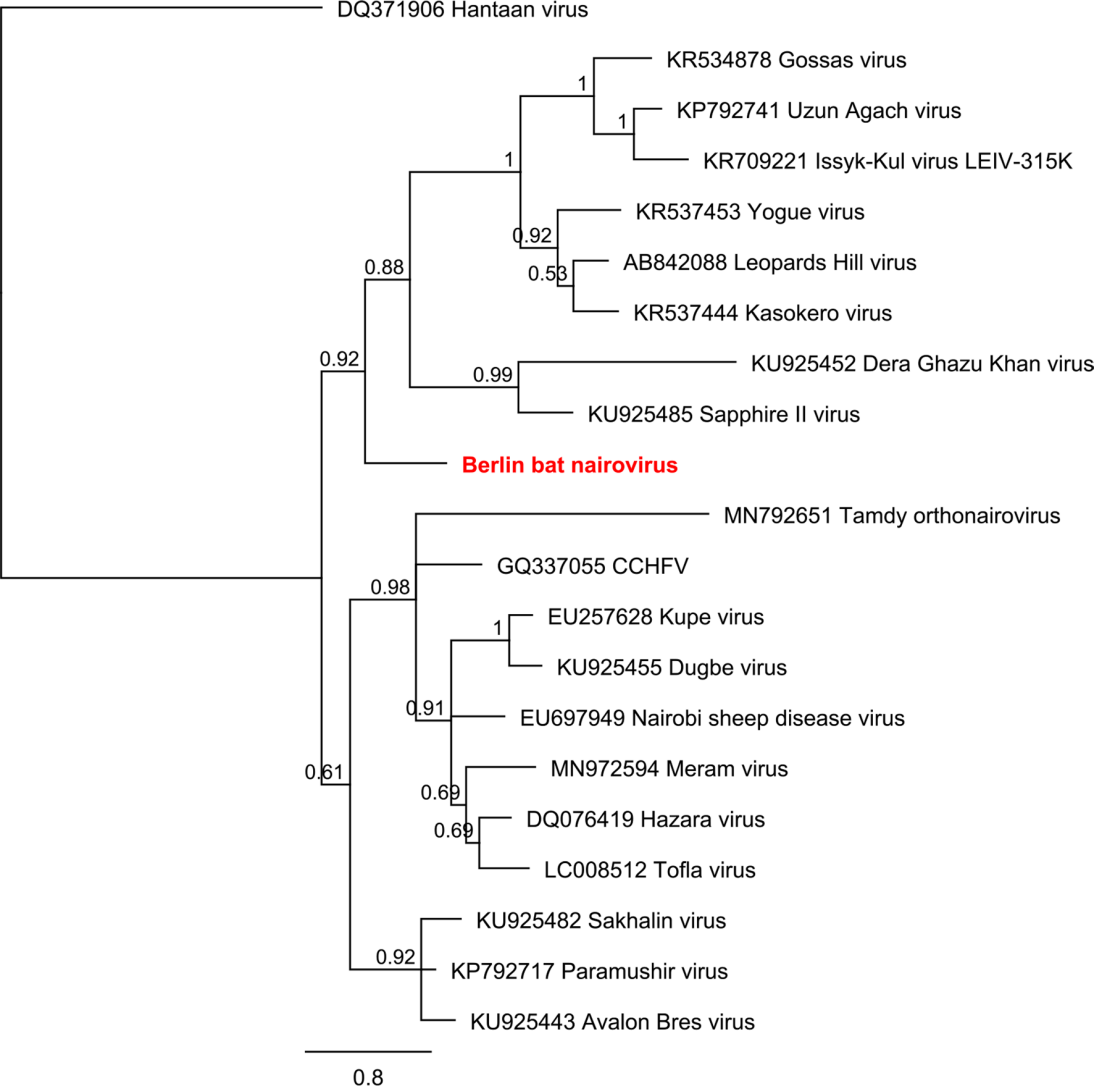
Phylogenetic reconstructions of Nairoviruses—2 ML, 1 million, 457 nt of the L segment. Reconstruction was performed via the Bayesian MCMC approach using MrBayes with the following settings (burn-in, 30%; frequency, 100; chain length, 1 million to 10 million, depending on when a standard derivation of below 0.025 was reached)^78^. Hantaan virus was used as an outgroup. Reconstructed trees were visualized using FigTree and posterior probabilities were depicted for each node (http://tree.bio.ed.ac.uk/software/figtree/).

**Figure 6:**
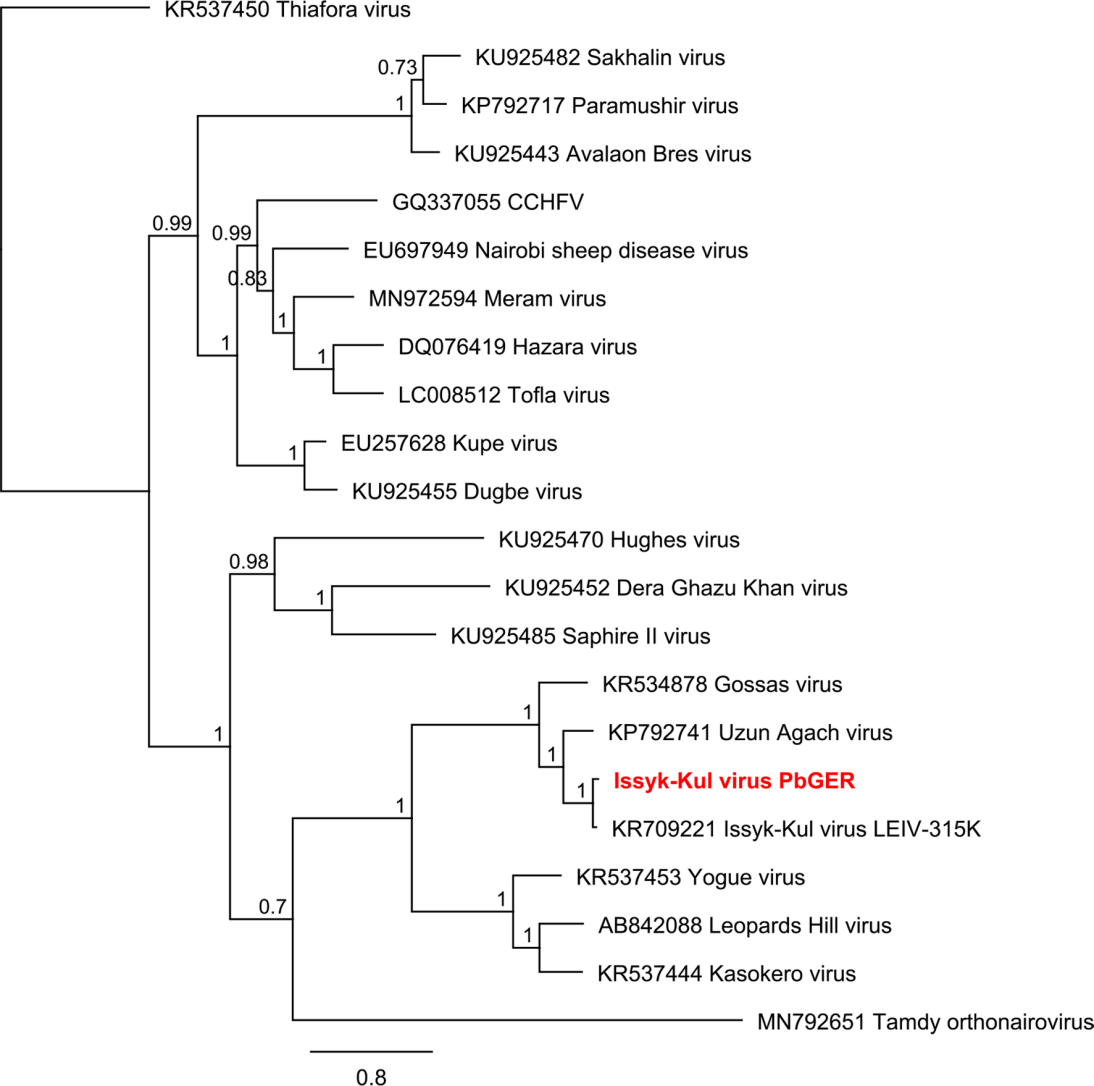
Phylogenetic reconstructions of Nairoviruses ML, 1 million, 1415 nt of the L segment. Reconstruction was performed via the Bayesian MCMC approach using MrBayes with the following settings (burn-in, 30%; frequency, 200; chain length, 1 million to 10 million, depending on when a standard derivation of below 0.025 was reached)^78^. Hantaan virus was used as an outgroup. Reconstructed trees were visualized using FigTree and posterior probabilities were depicted for each node (http://tree.bio.ed.ac.uk/software/figtree/).

Nairoviruses (n = 3) were found in pools 3, 5 and 6 (8132 reads). The found virus sequences share the highest identity with Issyk-Kul virus, Sapphire II virus and Avalon Bres virus. Obtained sequences are available under the following accession numbers: MN851301, MN851292 and MN851296. Phylogenetic reconstruction of Sapphire II-like virus and Issyk-Kul-like virus with other members of nairoviruses (457 nt, L-segment) is displayed in Figs. 5 and 6. Because of missing sequence homology between Avalon Bres virus-like virus with the other two strains, the phylogenetic reconstruction is not shown, but available on request.

#### Phenuiviridae

**Figure 7:**
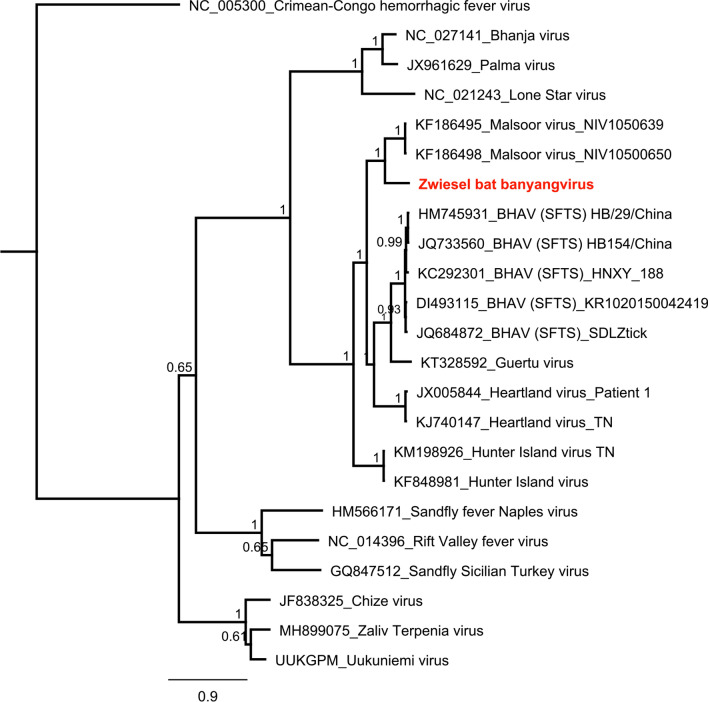
Phylogenetic reconstruction of Phenuiviruses glycoprotein–ML, 1 million, 2578 nt. Reconstruction was performed via the Bayesian MCMC approach using MrBayes with the following settings (burn-in, 30%; frequency, 100; chain length, 1 million to 10 million, depending on when a standard derivation of below 0.025 was reached)^78^. Reconstructed trees were visualized using FigTree and posterior probabilities were depicted for each node (http://tree.bio.ed.ac.uk/software/figtree/).

Phenuiviruses (n = 3) were found in pools 3 and 8 (8061 reads). The found virus sequences share the highest identity with the strains Laurel Lake virus (genus Laulavirus) (n = 2) and Malsoor virus/SFTS (n = 1) (genus Banyangvirus). Obtained sequences > 200 nt are available under the following accession numbers: MN851297 and MN857542. Phylogenetic reconstruction of Malsoor-like virus with other members of phenuiviruses (2578 nt, glycoprotein) is displayed in Fig. 7. Laurel Lake-like viruses 1 and 2 with other members of phenuiviruses is displayed in the supplemental section (Supplementary Fig. S3).

#### Tymovirus

Tymoviruses (n = 2) were found in pools 3 and 5 (423 reads). The found virus sequences share the highest identity with the strains Bombyx mori latent virus and Grapevine Red Globe virus. The virus sequences were named after the order of appearance and in relation to reference strains: Bombyx mori latent-like virus and Grapevine Red Globe-like virus. Obtained sequences are available under the following accession numbers: MN851298 and MN851299.

#### Retroviridae

Retroviruses (n = 5) were found in pools 1, 6 and 9 (234 reads). The found viral sequences share the highest identity with the strains *Myotis brandtii* endogenous retrovirus, Feline endogenous virus FERV1, and *Myotis lucifugus* endogenous retrovirus. The virus sequences were named after their host and in relation to reference strains: *Pipistrellus pipistrellus* endogenous retrovirus, *Myotis myotis* endogenous retrovirus, *Myotis daubentonii* endogenous retrovirus. Obtained sequences are available under the following accession numbers: MN851300, MN851302 and MN851303. Phylogenetic reconstructions for *Myotis brandtii* endogenous retrovirus-like sequence and *Myotis lucifugus* endogenous retrovirus-like sequence are available on request.

#### Phages

Numerous phages were found in all pools (226,696 reads). Most of the phages belong to the *Myoviridae* and *Podoviridae*, followed by the *Siphoviridae*.

### Comparison of PCR and virome results

Of all viruses identified by family-specific PCR in the original samples, none was found by metagenomic sequencing. Vice versa, none of the viruses found by the metagenomic virome approach was detected by family-specific PCRs targeting the same family. The initially isolated reovirus T3/Bat/Germany/342/08 was identified by virome sequencing within the original sample.

## Discussion

Virome sequencing of German bat carcasses of 16 bat species resulted in a high variety of confirmed viral sequences of parvoviruses, picornaviruses, totiviruses, reoviruses, nairoviruses, phenuiviruses, tymoviruses, retroviruses and several phages. In addition to the confirmed viral sequences, several sequencing reads were identified that shared a high homology and identity to other viruses. These high-identity reads were excluded as they were very likely false-positive results (i.e. poxviruses) (Fig. 1). The occurrence of false-positive results in virome sequencing is well known and has been described before^39^. Table 1 compares the results obtained from different bat virome studies with our findings. Virome profiles found in our study are generally comparable to those in other studies. Several Picornaviruses, Parvoviruses, Retroviruses, Tymoviruses and Totiviruses were identified in the nine distinct virome profiles of European bats (Supplementary Tables S3, S4, S5, S6, S7, S8, S9, S10 and S11). However, some of the viruses detected in our study are particularly interesting as they are phylogenetically closely related to viruses that can cause diseases in humans, or they are the first description of these viruses in certain bat species or within the European geographical range. The following discussion gives a summary of the viruses of highest interest. The full discussion on these viruses can be found in the supplemental section.

Six Bunyaviruses were identified in this study (nairoviruses and phenuiviruses). *Nairoviridae* are a family within the order *Bunyavirales* and named after the type species Nairobi sheep disease virus^40^. The majority of nairoviruses is transmitted by ticks and several are capable of causing severe diseases in humans and animals^40^. Bat nairoviruses have been reported before: Ahun nairovirus (KF170224) and Gossas virus (KR534878)^21,28,41^. Other nairoviruses of bats have been described and form two monophyletic genogroups within the nairoviruses, Keterah and Kasokero^41^.

In this study three novel nairoviral sequences were detected and confirmed in tissues from German bats; these are related to Issyk-Kul virus (Id 95% nt; 99% aa), Sapphire II virus (Id 85% nt; 54% aa) and Avalon Bres virus (Id 71% nt; 50% aa). Nine out of twelve *Eptesicus nilssonii* bats in pool 3 were infected with a yet undescribed Issyk-Kul virus strain (Supplementary Table S5). The phylogenetic reconstruction clearly allocated this novel strain to already described Issyk-Kul viruses within the Keterah genogroup (Fig. 6)^41^. Issyk-Kul virus had first been isolated in 1970 from a *Nyctalus noctula* bat in Kyrgyzstan and later on in Tajikistan and Kazakhstan^42,43^. Issyk-Kul virus was described to cause sporadic febrile outbreaks in humans with headache, myalgia and nausea^42,44^. It is assumed that Issyk-Kul virus can be transmitted by tick bites and exposure to bat feces and urine, eventually^42^. The Issyk-Kul-like virus described here was found predominantly in liver, spleen and lung tissues of the respective bats, indicating systemic infection of bats instead of solely passaging intestinal tick content. We named the novel Issyk-Kul virus strain “PbGER” after its origin in Prackenbach, Germany (Table 4). The novel Issyk-Kul virus strain PbGER (ISKV PbGER) is further analyzed by whole genome sequencing as well as throughout analysis; this is the subject of another study^45^. Our findings show for the first time the abundance of this virus in Europe and within this species.

Sapphire II-like virus was detected in eleven *Pipistrellus pipistrellus* bats from pool 5 and confirmed predominantly in lung and spleen tissues (Supplementary Table S7). Phylogenetic reconstruction indicated that Sapphire II-like virus is related to Sapphire II virus and clusters with the Dera Ghazi Khan genogroup usually associated with birds^41^ (Fig. 5). Sapphire II virus was isolated from swallowed ticks in 1972 and was not reported to cause any diseases in humans^46^. This is the first description of this genotype in bats. We named the Sapphire II-like virus Berlin bat nairovirus (BbnV) after the origin of the corresponding bats.

Avalon Bres virus-like sequence was detected in *Pipistrellus pipistrellus* bats of pool 6 (Supplementary Table S8). Phylogenetically, Avalon Bres virus clusters monophyletically with the Sakhalin genogroup. Viruses of these genogroups have not been described before to be associated with bats. However, a serological study showed that several wild-caught bats had antibody responses to CCHFV proteins^47^. We named the Avalon Bres virus-like virus Wittenau bat nairovirus (WbnV) after the origin of the corresponding bats.

Phenuivirus is a family within the order *Bunyavirales*^40^. The majority of the ten phenuivirus genera is mosquito-borne; however, some genera are transmitted by ticks (i.e. Banyangviruses) and are capable of causing severe diseases in humans and animals. Three phenuiviruses (genus phlebovirus) have been reported to be identified from bats: Malsoor virus, Rift Valley virus and Toscana virus^42,43^. Malsoor virus was isolated from *Rousettus leschenaultii* in India and is by phylogenetic reconstruction monophyletic with viruses of the genus Banyangvirus, being related to Huaiyangshan banyangvirus (former SFTS) and Heartland virus which are capable of causing severe diseases in humans^48^. Here we describe the detection of three phenuiviruses related to Laurel Lake virus (Id 43% aa) (genus Laulavirus) and Malsoor virus /SFTS (Id 70% nt; 72% aa) (genus Banyangvirus).

Six *Eptesicus nilssonii* bats from pool 3 were tested positive for Malsoor-like virus in liver, spleen, lungs and intestines (Supplementary Table S5). Phylogenetically, the Malsoor-like virus from *Eptesicus nilssonii* clusters monophyletically with the genus banyangvirus Huaiyangshan banyangvirus (SFTS) virus, Heartland virus and Malsoor virus (Fig. 7). This evolutionary distance could indicate a potential zoonotic transmission of both Malsoor virus and the found Malsoor-like virus to humans. We named the Malsoor-like virus Zwiesel bat banyangvirus (ZbbV) after the origin of the corresponding bats. The novel Zwiesel bat phlebovirus is further analyzed by whole genome sequencing as well as throughout analysis; this is the subject of another study published simultaneously as a spin-off to this study^49^.

Laurel Lake-like virus 1 and Laurel Lake-like virus 2 (genus Laulavirus) were identified in bats from pool 8 (Supplementary Table S10). Pool 8 comprises bats of two species, *Pipistrellus nathusii* and *Plecotus aureus*. Phylogenetic reconstruction of the Laurel Lake-like virus showed that Laurel Lake virus and Laurel Lake-like virus are quite distanced from the other viruses of the Uukuniemi group, although they are clearly clustering (Supplementary Fig. S3)^50^. We named the Laurel Lake-like virus 1 Bavarian bat lalavirus (BblV) after the origin of the corresponding bats. We named the Laurel Lake-like virus 2 Munich bat lalavirus (MblV) after the origin of the corresponding bats.

Three reovirus sequences (genera orbivirus, rotavirus and orthoreovirus) were identified in the virome data of pools 2, 5 and 8 (Supplementary Tables S4, S5 and S10). The orthoreovirus in pool 8 has been known before (T3/Bat/Germany/342/08) and served as a kind of positive control for this study, as we added the bat tissue from bat 342/08, from which the virus was initially isolated, to the pool^1^ (Supplementary Table S10). The orbivirus and rotavirus are genera within the subfamily *Sedoreovirinae* of the family *Reoviridae*. The orbiviruses comprise several species that are inducing severe diseases in humans and animals (i.e. bluetongue disease or epizootic hemorrhagic disease virus) and are capable of replication in several arthropod and vertebrate hosts^51^. In pool 2 an orbivirus related to the yet unpublished Bat orbivirus from China (AccNo. MH144554.1) (Id 81% aa) and Sathuvachari virus was identified in *Nyctalus noctula* bats (Supplementary Table S4) (Fig. 3). Sathuvachari virus has first been isolated in India in 1963, and because of its orbivirus character has tentatively been classified as mosquito-borne although the strain was isolated from a bird (starling)^52^. There have been two further orbiviruses identified from bats (*Myotis rickettsi* and *Rhinolphus ferrumequinum*) in China (Acc. No. KX343070.1 and KX161703.1). We named the Sathuvachari-like virus Irlbach bat orbivirus (IboV) after the origin of the corresponding bats.

The bat rotavirus identified in this study was detected in pool 5 which comprises tissues from *Pipistrellus pipistrellus* bats (Supplementary Table S7). Phylogenetic reconstruction allocates the bat rotavirus sequence into a distinct but related clade to rotaviruses type A (Id 58% nt; 57% aa) (Fig. 4). Numerous rotaviruses have been identified in bats but only one other rotavirus has been identified in European bats^28^. The bat rotavirus from France is similarly related to rotaviruses of group A. The zoonotic potential of these bat rotaviruses related to group A has yet to be determined. We named the rotavirus A-like virus Hannover bat rotavirus (HbrV) after the origin of the corresponding bats.

A sequence related to other members of the order *Mononegavirales* was identified in intestinal samples of *Nyctalus noctula* bats in pool 2 (Supplementary Table S4). It was described for these bats to have parasites in their intestines^53^; it is possible that the sequence originated from a tapeworm. On amino acid level the novel sequence shared highest similarity to Wenzhou tapeworm 1 virus (Id 35% aa) and Midway virus^54,55^. Phylogenetically the strain was grouped distinct from Midway virus and was more closely related to Borna disease virus and rabies virus. The similarities on nt level are quite low and the tree has to be interpreted with caution (SF2). However, these findings are interesting as bats are often discussed as potential reservoir host of many viruses of the order *Mononegavirales* (i.e. Ebola virus, Marburg virus, Nipah virus, Hendra virus and lyssaviruses). We named the Wenzhou tapeworm 1-like virus Berlin bat mononegavirus (BbmV) after the origin of the corresponding bats.

The screening of 375 bats with numerous family-specific PCRs resulted in several novel sequences (Table 2). The inoculation of the bat tissues into six different cell lines revealed two novel isolates (Supplementary Table S1). Virome sequencing of 189 histopathologically suspicious tissues of these bats resulted again in a high variety of novel virus sequences. Taking all of this into account, it is somewhat surprising to find different viruses in identical samples with these complementary approaches. However, the setup of the study design is insufficient to provide reliable statistics. For the RNA virus screening the organ material was homogenized in RNAlater® buffer and 30 µl per organ were pooled before extraction. For the DNA virus screening 30 µl of each organ natively frozen at − 80 °C was pooled before extraction. Cell culture isolation was also done with the native samples, although on differing cell culture systems. However, in cell culture only the occurrence of a visible CPE gives a clue of an ongoing virus infection and the absence of CPE does not exclude the potential infection. For the virome sequencing only those individual organs (native − 80 °C) were used that showed alterations in histopathology. If family-specific PCRs resulted in clear amplification and revealed a novel virus sequence after Sanger sequencing, one would expect to be able to obtain sequences of the same virus by virome sequencing of the same sample. In fact, many of the organ bat tissues that were prepared for virome sequencing also revealed positive results in family-specific PCRs. For example, Bat E95/09 (Supplementary Table S7) was tested positive for paramyxoviruses by PCR^32^. The respective tissues were subjected to virus purification and virome sequencing within pool 4, but no paramyxoviruses were detected in the virome data. A possible explanation could be that the purification method we have used, TUViD-VM, did not sufficiently purify paramyxoviruses from tissues; but this is exactly what this protocol was designed for^10^. Actually, the whole protocol was validated and tested with a range of viruses (i.e. paramyxovirus, poxvirus, influenza virus and reovirus), with the most remarkable results from paramyxovirus (Sendai virus)^10^. In addition, we have tested the reproducibility and consistency of the TUViD-VM protocol numerous times and found it to be very stable^56^. Furthermore, it has to be considered that we have used pooled tissues for our examinations instead of i.e. single organs. It is possible that this may have affected the detectability of the different viruses. However, we have used pooled tissues for all methods compared and therefore results should be comparable. Taking this into account, we propose that nothing is more important in virome studies than the ratio between targeted and untargeted sequences. For instance, if we apply a family-specific PCR to a mixture of cDNA containing 100 genome equivalents of the targeted paramyxovirus, we will most probably be able to obtain a clean and sufficient amplification of the targeted sequences, although the cDNA contains only the 100 genome equivalents of paramyxovirus over millions of other sequences. In comparison, the approach of virome sequencing differs significantly. When millions of other sequences compete (e.g. host genome) the likelihood of detection of an underrepresented number of viral sequences would be very low. To address and circumvent this obvious fact, the TUViD-VM protocol was applied in this study, as it aims to decrease the amount of background (host) sequences and amplify the viral sequences to increase the detection ratio and likelihood. One accompanying effect we observed was that high numbers of viral reads of other viruses (i.e. more than 900,000 reads for picornaviruses in pool 5) impeded the sequencing of low amounts of other viral amplicons. Similarly, the analyses of PCR-negative results revealed a surprising outcome. All bats were screened for the presence of bunyaviral sequences, especially for phenuiviruses and nairoviruses, by family-specific PCR with negative results. Interestingly, virome sequencing revealed the presence of six novel strains of bunyaviruses with identities ranging from 70 to 95 percent identity on nt level to known virus sequences.

This leads to the conclusion that the outcome of virus discovery studies, whether approached by PCR, metagenomics or cell culture, provides only a fraction of the viruses truly present in a sample. The methods should be used complementarily to give a more complete picture of virus diversity rather than to replace each other—as has often been described for the advantages of metagenomics over classic PCR approaches. The combined data of existing individual studies investigated by different virus discovery protocols with their conclusions are further used to extrapolate and predict outbreak risks and hotspots. As long as we use single virus detection protocols in studies only, the very true diversity of viruses will stay hidden, unrecognized by our insufficient methodological approaches. It might be time for common approaches, where scientists doing virus discovery and modeling work join forces and design studies in a more comparable and meaningful manner.

## Methods

### Study

**Figure 8:**
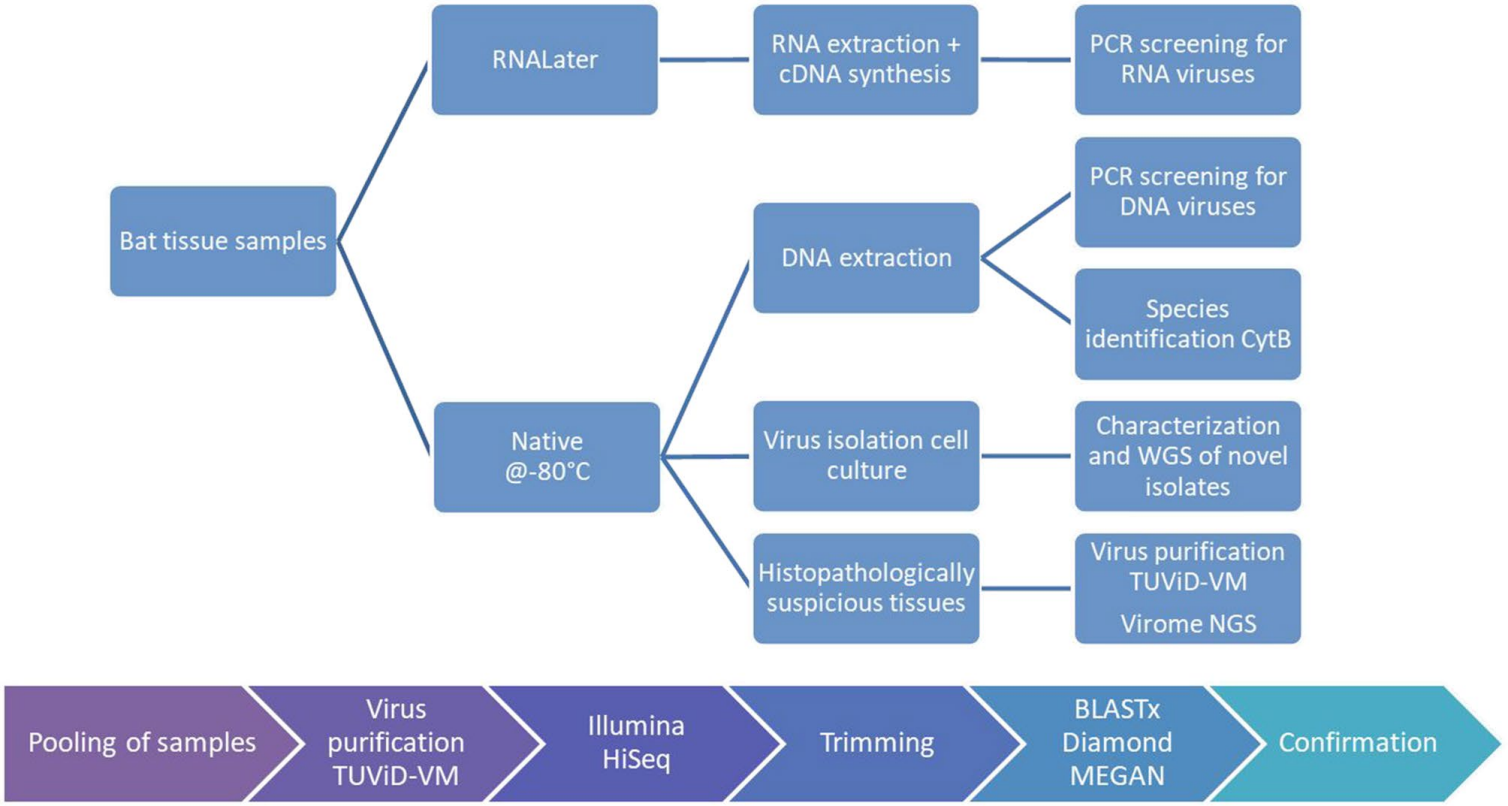
Workflow. The workflow of sample preparation and analysis is depicted.

In compliance with the species protection through the European Commission (https://ec.europa.eu/environment/nature/legislation/habitatsdirective/) and the Agreement on the Conservation of Populations of European Bats (www.eurobats.org), investigative research on bats was permitted by local government bodies^38^. We named the manuscript Virome of German Bats as the bats were found in Germany. However, bats are migrating animals, many of them covering long distances throughout Europe. Therefore, our results could also be expected in other parts of Europe. We herewith confirm that all permits to investigate carcasses of deceased bats and all experimental protocols on the dead bats were approved by the respective local governmental authorities (district government of Upper Bavaria, Munich [No. 55.1-8642.1-4-2006]; district government of Bavarian Swabia, Augsburg [No. 51-8645.11/489]; Lower Saxony water management, coastal defense and nature conservation, Hanover [No. 897.21-20] and senate department for urban development and the environment, Berlin [No. I E 222-10.04.2004]). We herewith further confirm that all experiments were performed in accordance with relevant guidelines and regulations. As part of a study on diseases in native bats^38^, 375 bats of 18 vespertilionid bat species were examined (*Myotis mystacinus* [n = 37], *M. daubentonii* [n = 17], *M. bechsteinii* [n = 1], *M. brandtii* [n = 2], *M. myotis* [n = 5], *M. nattereri* [n = 9], *Pipistrellus pipistrellus* [n = 9698], *P. nathusii* [n = 28], *P. kuhlii* [n = 8], *P. pygmaeus* [n = 3], *Vespertilio murinus* [n = 20], *Barbastella barbastellus* [n = 2], *Plecotus austriacus* [n = 1], *Plecotus auritus* [n = 20], *Eptesicus nilssonii* [n = 15], *E. serotinus* [n = 20], *Nyctalus noctula* [n = 86] and *N. leisleri* [n = 3]). Animals were found dead, injured or moribund near roosting sites or human habitations^38^ in urban and suburban areas of different regions in Italy (n = 1) and Germany (n = 374) (Bavaria [n = 150], Lower Saxony [n = 35] and Berlin greater metropolitan area [n = 148], Brandenburg [n = 36], Baden Wuerttemberg [n = 3]). All bat carcasses were kindly provided by bat researchers and bat rehabilitation centers from the different geographic regions. In case bats died in care or had to be euthanized for medical reasons, the carcasses were handled as described before^38,57–59^. Aliquots of the individual organs were divided between two tubes: one tube with RNAlater® (Qiagen, Hilden, Germany) for RNA extraction and one tube native frozen at − 80 °C and sent to the Robert Koch Institute for virological examination (Fig. 8).

All work was performed at Biosafety level-2 conditions with appropriate precautions. The study was divided into three methodological sections. (1) PCR screening, (2) Virus isolation via cell culture and (3) Virome analysis. Every section had its own difficulties and individual steps for sample preparation explained in the following.

#### PCR screening for RNA viruses

30 µl of each of the eight individual organ homogenates (in RNAlater®) of one respective bat were pooled (lungs, liver, spleen, heart, brain, salivary gland, intestine and kidney). RNA extraction from RNAlater®-stabilized samples and cDNA synthesis was performed as described before^32^. The pool containing 240 µl of organ homogenate was then extracted using the PureLinkTM Viral RNA/DNA Mini Kit (Invitrogen, Carlsbad, USA). Extracted RNA was transcribed to cDNA by using the TaqMan® Reverse Transcription Reagents (Applied Biosystems, Darmstadt, Germany). The cDNA pools of 375 bats were screened by differing family-specific PCR systems for the detection of paramyxoviruses^60^, arenaviruses^61^, coronaviruses^35^, filoviruses^62^, flaviviruses^63^, hantaviruses^64^, nairo- and phleboviruses^65^ and influenza viruses^66^.

#### PCR screening for DNA viruses

30 µl of each of the eight individual organ homogenates (native frozen at − 80 °C) of one respective bat were pooled (lungs, liver, spleen, heart, brain, salivary gland, intestine, kidney). DNA was extracted using the PureLinkTM Viral RNA/DNA Mini Kit (Invitrogen) as described before^31,33^. The DNA pools of 375 bats were screened by differing family-specific PCR systems for the detection of adenoviruses^67^, herpesviruses^68^ and poxviruses^69,70^.

#### Virus isolation in cell culture

Virus isolation in cell culture was performed from pools containing all eight individual organs of the respective bats natively frozen at − 80 °C as described before^30,31^. For cell culture isolation different cell culture systems were used; these are listed in Supplementary Table S1.

#### Virome sequencing

**Figure 9:**
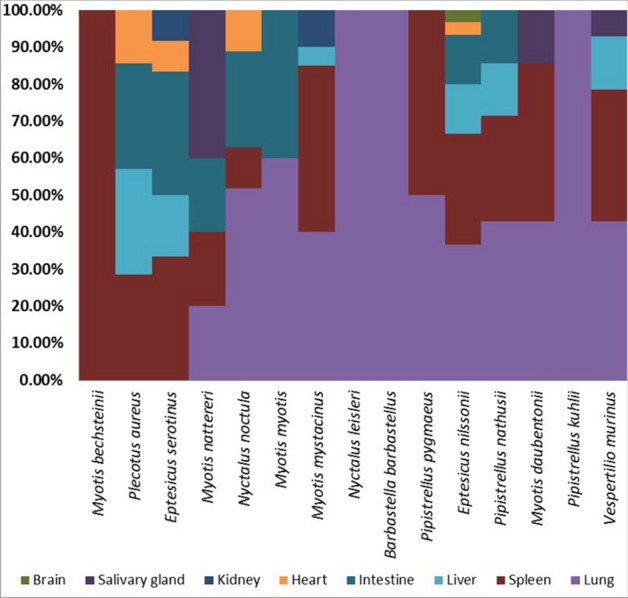
Percentage of individual organs pooled per species is depicted.

Selected single organs (native frozen at − 80 °C) of 118 of the 375 individual bats were subjected to further investigation. These 118 bats and their corresponding 189 native frozen organ tissues were chosen by analysis of the pathological findings^71^. Several bats were found to have alterations that may be related to viral infections (e.g., interstitial pneumonia, pulmonary BALT hyperplasia, infiltrates of mononuclear cells in different organs, diffuse enlarged villi and mononuclear intestinal cell infiltrates or catarrhal or hemorrhagic enteritis). Due to the large number of individual samples, organs were allocated to nine pools for virus purification with TUViD-VM and sequencing^10^. Pools were established using the following criteria: (a) Pools should consist of similar sample numbers, (b) Pools should have only one bat species and (c) If number of bats is not sufficient to build a single pool, species are mixed. Table 5 and Fig. 9 summarize the composition of these nine individual pools.

**Table 5:** Composition of bat pools.

Pool	# bats	Species (individuals per species)	Organs
1	12	*Myotis bechsteinii* (1)	S
		*Myotis nattereri* (3)	L, I, S, **SG**
		*Myotis myotis* (3)	L, I
		*Nyctalus leisleri* (1)	L
		*Barbastella barbastellus* (1)	L
		*Pipistrellus kuhlii* (3)	L
2	22	*Nyctalus noctula* (22)	**L**, **I**, S, H,
3	12	*Eptesicus nilssonii* (12)	**L**, I, **S**, H, Li, B
4	11	*Myotis mystacinus* (11)	**L**, **S**, Li, K
5	12	*Pipistrellus pipistrellus* (12)	**L**, I, **S**, Li, K
6	24	*Pipistrellus pipistrellus* (23)	**L**, I, S, SG, H, Li, K
		*Pipistrellus pygmaeus* (1)	L, **S**
7	6	*Eptesicus serotinus* (6)	**L**, **I**, **S**, H, Li, K
8	11	*Plecotus aureus* (7)	**L**, I, S, H, Li
		*Pipistrellus nathusii* (4)	**L**, I, S, Li
9	11	*Myotis daubentonii* (5)	L, **S**, SG
		*Vespertilio murinus *(6)	**L**, **S**, SG, Li
$\Sigma$	118	16 species	189 organs

Table summarizes species and organs for the nine individual pools used for metagenomics sequencing and cell culture isolation. Organs written in bold have the highest percentage in the individual pool.

# number, *L* lung, *I* intestine, *S* spleen, *SG* salivary gland, *H* heart, *Li* liver, *K* kidney, *B* brain.

200 µl of each individual organ from the 118 bats was pooled into nine differing pools, depending on histopathological results and according to the species. The composition of the individual pools is given in Table 5. Each of the nine pools was divided for inoculation onto Vero and Paki cell cultures for virus isolation as described before^72,73^. The original TUViD-VM purification protocol used in this study comprises the following steps: (1) homogenization, (2) clearing centrifugation, (3) ultracentrifugation through sucrose cushion, (4) ultracentrifugation, (5) digestion, (6) extraction, (7) cDNA and double strand synthesis and (8) random amplification and size selection via agarose gel electrophoresis^10^. Instead of using 200 µl of tissue homogenate as prescribed in the original protocol, 3 ml of organ tissue was used and the protocol was upscaled with small modifications^10^: in most pools the homogenate volume exceeded 3 ml; however, in case the amount of homogenate was too low, the volume was adapted with PBS to 3 ml; for ultracentrifugation, the rotors and pace were adapted accordingly by using the SW32 rotor (Beckman Coulter, Krefeld, Germany) also for the first ultracentrifugation step. From step (4) of the original TUViD-VM protocol onward, the protocol was performed without any further modifications.

After size selection by excision of the area between 500 and 1000 nt following agarose gel electrophoresis, samples were prepared for sequencing. Libraries were built using the NexteraXT protocol as described before^10^. The sequencing reaction was performed on the Illumina HiSeq Sequencer in high throughput mode using 150 cycles.

Raw data was processed as follows (Fig. 8): Adapters were trimmed and filtering of read length and quality was performed. Raw reads were then subtractive mapped against customized databases to remove background reads (bacteria and mammalian genomes). Remaining reads were assembled to contigs using Velvet^74^. Contigs were then compared to the viral protein database (nr NCBI) via the DIAMOND tool and results were visualized in MEGAN6 (–sensitive)^37,38^. Selected results from MEGAN6 were blasted using BLASTx on the NCBI GenBank database. Full genomes (if available) of the most similar sequences were downloaded to serve as a reference. The initial trimmed reads were then mapped to the reference sequence using Bowtie2 to identify more matching reads and check the correct allocation visually for quality^75^. Virus genomes were also assembled using Bowtie2. Sequences were checked for plausibility by analyzing the closest related virus. In the case of i.e. retroviruses the closest related sequences were also from bat species, proving that they are possible to occur. Sequences of highest interest were extended by spanning PCRs and primer walking followed by Sanger sequencing. For all sequences of interest specific primers were designed to confirm the sequences in cDNA by conventional PCR of the individual extracted pools and organ samples eventually. Cycling conditions and primers are available on request. Any bands of targeted height were purified, Sanger sequenced and compared to the original sequence obtained from Next Generation Sequencing (NGS).

Final sequences obtained from data analysis and Sanger sequencing were used to reconstruct phylogenetic trees. Sequences were aligned to type species (whenever possible) of the respective viral family (ICTV database) and other related viruses of interest via ClustalW. Alignment quality was checked with the online tool T-Coffee^76^. Model of evolution was predicted via jmodeltest and the model with the best AIC score was picked^77^. The actual reconstruction was performed via the Bayesian MCMC approach using MrBayes with the following settings (burn-in, 30%; frequency, 100; chain length, 1 million to 10 million depending on when a standard derivation of below 0.025 was reached)^78^. Reconstructed trees were visualized using FigTree and posterior probabilities were depicted for each node (http://tree.bio.ed.ac.uk/software/figtree/).

## Supplementary Information

Supplementary Information.
