# Supplementary material for: The virome of German bats: comparing virus discovery approaches
Source: Sci Rep. 2021 Apr 1;11:7430. doi: 10.1038/s41598-021-86435-4 (PMC8016945; doi:10.1038/s41598-021-86435-4)
Supplement: Supplementary file 1 — Supplementary Information. [file 41598_2021_86435_MOESM1_ESM.docx]

**The virome of German bats – comparing virus discovery approaches.**

Claudia Kohl^1#^, Annika Brinkmann^1^, Aleksandar Radonić^2^, Piotr Wojtek Dabrowski^3^, Kristin Mühldorfer^4^, Andreas Nitsche^1^, Gudrun Wibbelt^4^, Andreas Kurth^1^

^1^Robert Koch Institute, Centre for Biological Threats and Special Pathogens, Berlin, Germany

^2^Robert Koch Institute, Methodology and Research Infrastructure 2, Genome Sequencing, Berlin, Germany

^3^Robert Koch Institute, Methodology and Research Infrastructure 1, Bioinformatics, Berlin, Germany

^4^Leibniz Institute for Zoo and Wildlife Research, Berlin, Germany

^#^corresponding author:

Dr. Claudia Kohl

Centre for Biological Threats and Special Pathogens
Robert Koch Institute
Seestrasse 10

13353 Berlin, Germany

kohlc@rki.de

**Results (Full)**

**Virome Sequencing and Phylogenetic Reconstruction**

Sequencing of the nine bat pools via Illumina HiSeq revealed 127,338,644 reads after trimming. In Figure 1 the raw output obtained from MEGAN6^1^ is depicted. Though MEGAN6 displayed a large number of hits for different viral families, several hits did not meet the quality criteria. These reads could not be remapped to reference sequences, revealed non-plausible BLASTx/BLASTn results (i.e. genomic DNA of bats) or shared 100 percent identity to sequences obtained before (possible cross-contamination). Supplementary Table S2 summarizes the number of filtered reads (length/quality) obtained per pool with the number of viral reads (w/o phages) finally checked for quality and allocated by Diamond^2^, MEGAN6 and further analysis. In Figure 2 the overall number of viral reads in all pools per virus family is summarized. Viruses of nine different families and orders (*Parvoviridae*, *Picornaviridae*, *Totiviridae*, *Mononegavirales*, *Reoviridae*, *Bunyavirales*, *Tymovirus*, *Retroviridae* and phages) were confirmed within the bat samples by virome sequencing and further analysis. For each family, selected viruses were retested by specific PCRs in the individual bats’ organs. For the viruses of highest interest, phylogenetic reconstructions were calculated as described in the methods section.

***Parvoviridae***

Parvoviruses (n=5) were found in pools 1, 2, 3 and 9 belonging to the subspecies *Densovirinae* (146,313 reads) and *Bufavirinae* (20,296 reads). The virus sequences were named after the order of appearance and in relation to reference strains. Blatella Germanica densovirus-like virus 1, Blatella Germanica densovirus-like virus 2, Blatella Germanica densovirus-like virus 3, Parus major densovirus-like virus and Bat bufavirus were confirmed by sequence assembly and comparison to reference strains and to each other as described in the methods section. Pools 1 and 3 were tested back for Blatella Germanica densoviruses with designed specific primers. Blatella Germanica densovirus-like 1 virus was confirmed by PCR and Sanger sequencing in pool 1, and Blatella Germanica densovirus-like 2 virus was confirmed in pool 3. The individual length of obtained contigs, the accession number of the reference sequence and the pairwise identity on nt and aa level are summarized in Table 3. Accession numbers of obtained sequences are available in Table 4.

***Picornaviridae***

Picornaviruses (n=7) were found in pools 1, 2, 3, 4, 5, 7 and 8 (1,039,379 reads), all of which were related to King virus UWV1, King virus UWV2, Tetnovirus 1 or Hubei picorna-like virus. All seven strains were confirmed by sequence assembly and comparison to reference strains and to each other as described in the methods section. Tetnovirus 1–like virus was tested in pool 2 and pool 4 and Hubei picorna-like virus 1 in pool 8. Pools 2 and 4 were tested back for Tetnovirus 1–like virus with designed specific primers and Hubei picorna-like virus 1 was tested back in pool 8. Tetnovirus 1–like viruses and Hubei picorna-like virus 1 were confirmed by PCR and Sanger sequencing in the corresponding pools. The individual length of obtained contigs, the accession number of the reference sequence and the pairwise identity on nt and aa level are summarized in Table 3. Obtained sequences are available in Table 4.

***Totiviridae***

One totivirus was found in pool 2 (881,349 reads) which appears to be most closely related to Eimeria Tenella RNA virus 1. The strain was confirmed by sequence assembly and comparison to a reference strain as described in the methods section. The individual length of obtained contigs, the accession number of the reference sequence and the pairwise identity on nt and aa level are summarized in Table 3. Obtained sequences are available in Table 4.

**Mononegavirus**

A distinct sequence, related to mononegaviruses Wenzhou tapeworm virus and Midway virus, was identified in pool 2 (14,628 reads). The potential strain was confirmed by sequence assembly and comparison to the reference strains as described in the methods section. Bats of pool 2 were tested back for Wenzhou tapeworm virus-like virus with designed specific primers and confirmed by PCR and Sanger sequencing in lung tissues of four individual bats. The individual length of obtained contigs, the accession number of the reference sequence and the pairwise identity on nt and aa level are summarized in Table 3. Obtained sequences are available in Table 4. Phylogenetic reconstruction of 665 nt (polymerase) Wenzhou tapeworm virus-like virus in comparison to the reference strains of *Mononegavirales* is displayed in (Supplementary Figure S2).

***Reoviridae***

Reoviruses (n=3) were found in pools 2, 5 and 8 (97,161 reads) and include related reoviruses of three distinct genera orbivirus (Bat orbivirus China – unpublished AccNo MH144554.1), rotavirus (Human rotavirus A-like virus) and orthoreovirus (Bat orthoreovirus T3/Bat/Germany/342/08). Bat orthoreovirus T3/Bat/Germany/342/08 had been isolated before from the same sample set; no further analysis was conducted here^30^. However, all three strains were confirmed by sequence assembly and comparison to reference strains and to each other as described in the methods section. The individual length of obtained contigs, the accession number of the reference sequence and the pairwise identity on nt and aa level are summarized in Table 3. Obtained sequences are available in Table 4. Phylogenetic reconstruction of orbiviruses (780 nt) and rotaviruses (450 nt, VP4) in comparison to the two novel sequences is displayed in Figures 3 and 4, respectively.

***Nairoviridae***

Nairoviruses (n=3) were found in pools 3, 5 and 6 (8,132 reads). The found virus sequences share the highest identity with strains Issyk-Kul virus, Sapphire II virus and Avalon Bres virus. All three strains were confirmed by sequence assembly and by comparison to reference strains and to each other as described in the methods section. Bats of pool 5 were tested for Sapphire II-like virus and confirmed by PCR in eleven individuals. Additionally, bats of pool 3 were tested back for Issyk-Kul-like virus with designed specific primers and confirmed by PCR and Sanger sequencing in tissues of nine individual bats. The individual length of obtained contigs, the accession number of the reference sequence and the pairwise identity on nt and aa level are summarized in Table 3. Obtained sequences are available in Table 4. Phylogenetic reconstruction of Sapphire II-like virus and Issyk-Kul-like virus with other members of nairoviruses (410 nt, L-segment) is displayed in Figure 6. Because of missing sequence homology between Avalon Bres virus with the other two strains, the phylogenetic reconstruction is not shown.

***Phenuiviridae***

Phenuiviruses (n=3) were found in pools 3 and 8 (8,061 reads). The found virus sequences share the highest identity with the strains Laurel Lake virus (genus Laulavirus) (n=2) and Malsoor virus/SFTS (n=1) (genus Banyangvirus). All three strains were confirmed by sequence assembly and comparison to reference strains and to each other as described in the methods section. Additionally, six individual *Eptesicus nilssonii* bats of pool 3 were tested positive for Malsoor-like virus by designed specific primers and PCR and confirmed by Sanger sequencing^39^. The individual length of obtained contigs, the accession number of the reference sequence and the pairwise identity on nt and aa level are summarized in Table 3. Obtained sequences are available in Table 4. Phylogenetic reconstruction of Malsoor-like virus with other members of phenuiviruses (2,578 nt, glycoprotein) is displayed in Figure 7. Laurel Lake-like virus 1 and 2 with other members of phenuiviruses is displayed in the supplemental section (Supplementary Figure S3).

**Tymovirus**

Tymoviruses (n=2) were found in pools 3 and 5 (423 reads). The found virus sequences share the highest identity with the strains Bombyx mori latent virus and Grapevine Red Globe virus. Both strains were confirmed by sequence assembly and comparison to reference strains and to each other as described in the methods section. The individual length of obtained contigs, the accession number of the reference sequence and the pairwise identity on nt and aa level are summarized in Table 3. Obtained sequences are available in Table 4.

***Retroviridae***

Retroviruses (n=5) were found in pools 1, 6, and 9 (234 reads). The found viral sequences share the highest identity with the strains Myotis brandtii endogenous retrovirus, Feline endogenous virus FERV1, and Myotis lucifugus endogenous retrovirus. All strains were confirmed by sequence assembly and comparison to reference strains and to each other as described in the methods section. Phylogenetic reconstructions for Myotis brandtii endogenous retrovirus-like sequence and Myotis lucifugus endogenous retrovirus-like sequence are available on request. The individual length of obtained contigs, the accession number of the reference sequence and the pairwise identity on nt and aa level are summarized in Table 3. Obtained sequences are available in Table 4.

**Phages**

Numerous phages were found in all pools (226,696 reads). Most of the phages belong to the *Myoviridae* and *Podoviridae*, followed by the *Siphoviridae*.

**Discussion of viruses found (full)**

Virome sequencing of European bat carcasses of 16 bat species resulted in a high variety of confirmed viral sequences of parvoviruses, picornaviruses, totiviruses, reoviruses, nairoviruses, phenuiviruses, tymoviruses, retroviruses and several phages. In addition to the confirmed viral sequences, several sequencing reads were identified that shared a high homology and identity to other viruses. These high-identity reads were excluded as they are very likely false-positive results (i.e. poxviruses) (Figure 1). The occurrence of false-positive results in virome sequencing is well known and has been described before^3^. Table 1 compares the results obtained from different bat virome studies with our findings. Virome profiles found in our study are generally comparable to those in other studies. Several picornaviruses, parvoviruses, retroviruses, tymoviruses and totiviruses were identified in the nine distinct virome profiles of European bats (Supplementary Tables S3, S4, S5, S6, S7, S8, S9, S10 and S11). However, some of the viruses detected in our study are particularly interesting as they are closely related phylogenetically to viruses that can cause diseases in humans, or they are the first description of these viruses in certain bat species or within the European geographical range. The following discussion focusses on the viruses of highest interest.

Six bunyaviruses were identified in this study (nairoviruses and phenuiviruses). *Nairoviridae* are a family within the order *Bunyavirales* and named after the type species Nairobi sheep disease virus^4^. The majority of nairoviruses is transmitted by ticks and several are capable of causing severe diseases in humans and animals^4^. A bat nairovirus, Ahun nairovirus (KF170224), has been detected earlier in lung tissues of one *Pipistrellus pipistrellus* and one *Myotis mystacinus*; phylogenetically Ahun nairovirus appears as a new clade, distinct from other nairoviruses^5, 6^. Another potential bat nairovirus (Gossas virus KR534878), identified by metagenomics before, originates from fecal samples of *Molossus molossus* bats from French Guiana^7^. The sequence was phylogenetically analyzed in the original study and has clearly clustered within the genus phlebovirus, though the authors describe the virus as nairovirus^7^. However, more nairoviruses of bats have been described and form two monophyletic genogroups within the nairoviruses, Keterah and Kasokero^6^. In this study three novel nairoviral sequences were detected and confirmed in tissues from European bats; these are related to Issyk-Kul virus (Id 95% nt; 99% aa), Sapphire II virus (Id 85% nt; 54% aa) and Avalon Bres virus (Id 72% nt; 50% aa). Nine out of twelve *Eptesicus nilssonii* bats in pool 3 were infected with a yet undescribed Issyk-Kul-like nairovirus (Supplementary Table S5). The phylogenetic reconstruction clearly allocated this novel strain to already described Issyk-Kul viruses within the Keterah genogroup (Figure 5)^8^. Issyk-Kul virus had first been isolated in 1970 from a *Nyctalus noctula* bat in Kyrgyzstan and later on in Tajikistan and Kazakhstan^9, 10^. Issyk-Kul virus was described to cause sporadic febrile outbreaks in humans with headache, myalgia and nausea^10^. It is assumed that Issyk-Kul virus can be transmitted by tick bites and exposure to bat feces and urine, eventually^10^. The Issyk-Kul-like virus described here was found predominantly in liver, spleen and lung tissues of the respective bats, indicating systemic infection of bats instead of solely passaging intestinal tick content. We named the Issyk-Kul-like virus Issyk-Kul-virus strain Prackenbach (PbGER) after the origin of the corresponding bats (Table 5). The novel Prackenbach bat nairovirus is further analyzed by whole genome sequencing as well as throughout analysis; this is the subject of another study, published simultaneously as a spin-off to this study^11^. Our findings show for the first time the abundance of this virus in Europe and within this species.

Sapphire II-like virus was detected in eleven *Pipistrellus pipistrellus* bats from pool 5 and confirmed predominantly in lung and spleen tissues (Supplementary Table S7). Phylogenetic reconstruction indicated that Sapphire II-like virus is related to Sapphire II virus and clusters with the Dera Ghazi Khan genogroup usually associated with birds^8^ (Figure 6). Sapphire II virus was isolated from swallowed ticks in 1972 and was not reported to cause any diseases in humans^12^. This is the first description of this genotype in bats. We named the Sapphire II-like virus Berlin bat nairovirus (BbnV) after the origin of the corresponding bats.

Avalon Bres virus like sequence was detected in *Pipistrellus pipistrellus* bats of pool 6 (Table S8). Phylogenetically, Avalon Bres virus clusters monophyletically with the Sakhalin genogroup. Viruses of these genogroups have not been described before to be associated with bats. However, a serological study showed that several wild-caught bats had antibody responses to CCHFV proteins^13^. We named the Avalon Bres-like virus Wittenau bat nairovirus (WbnV) after the origin of the corresponding bats. Phenuivirus is a family within the order *Bunyavirales* (4). The majority of the ten phenuivirus genera is mosquito-borne; however, some genera are transmitted by ticks (i.e. Banyangviruses) and are capable of causing severe diseases in humans and animals. Three phenuiviruses (genus phlebovirus) have been reported to be identified from bats: Malsoor virus, Rift Valley virus and Toscana virus^14-16^. Malsoor virus was isolated from *Rousettus leschenaultii* in India and is by phylogenetic reconstruction monophyletic with viruses of the genus Banyangvirus, being related to Huaiyangshan banyangvirus (former SFTS) and Heartland virus which are capable of causing severe diseases in humans^14^. Rift Valley virus was isolated from Pteropid bats in Guinea^16^ and Toscana virus from the brain of one *Pipistrellus kuhlii* in Italy, although doubts have arisen that this early finding might be due to a possible cross-contamination. Here we describe the detection of three phenuiviruses related to Laurel Lake virus (Id 43% aa) (genus Laulavirus) and Malsoor virus /SFTS (Id 70% nt; 72% aa) (genus Banyangvirus).

Six *Eptesicus nilssonii* bats from pool 3 were tested positive for Malsoor-like virus in liver, spleen, lungs and intestines (Supplementary Table S5). Phylogenetically, the Malsoor-like virus from *Eptesicus nilssonii* clusters monophyletically with the genus banyangvirus Huaiyangshan banyangvirus (SFTS) virus, Heartland virus and Malsoor virus (Figure 7). This evolutionary distance could indicate a potential zoonotic transmission of both Malsoor virus and the found Malsoor-like virus to humans. We named the Malsoor-like virus Zwiesel bat phlebovirus (ZbpV) after the origin of the corresponding bats. The novel Zwiesel bat phlebovirus is further analyzed by whole genome sequencing as well as throughout analysis; this is the subject of another study published simultaneously as a spin-off to this study^17^.

Laurel Lake-like virus 1 and Laurel Lake-like virus 2 (genus Laulavirus) were identified in bats from pool 8 (Supplementary Table S10). Pool 8 comprises bats of two species, *Pipistrellus nathusii* and *Plecotus aureus*. Phylogenetic reconstruction of the Laurel Lake-like virus showed that Laurel Lake virus and Laurel Lake-like virus are quite distanced from the other viruses of the Uukuniemi group, although they are clearly clustering (Supplementary Figure S3)^18^. We named the Laurel Lake like virus 1 Bavarian bat lalavirus (BblV) after the origin of the corresponding bats. We named the Laurel Lake-like virus 2 Munich bat lalavirus (MblV) after the origin of the corresponding bats.

Three reovirus sequences (genera orbivirus, rotavirus and orthoreovirus) were identified in the virome data of pools 2, 5 and 8 (Supplementary Tables S4, S7 and S10). The orthoreovirus in pool 8 has been known before (T3/Bat/Germany/342/08) and served as a kind of positive control for this study, as we added the bat tissue from bat 342/08, from which the virus was initially isolated, to the pool^1^ (Supplementary Table S10). The orbiviruses and rotaviruses are genera within the subfamily *Sedoreovirinae* of the family *Reoviridae*. The orbiviruses comprise several species that are inducing severe diseases in humans and animals (i.e. Bluetongue disease virus or epizootic hemorrhagic disease virus) and are capable of replication in several arthropod and vertebrate hosts^19^. In pool 2 an orbivirus related to the yet unpublished Bat orbivirus from China (AccNo. MH144554.1) (Id 81% aa) and Sathuvachari virus was identified in *Nyctalus noctula* bats (Supplementary Table S4). Sathuvachari virus has first been isolated in India in 1963 and, because of its orbivirus character, has tentatively been classified as mosquito-borne although the strain was isolated from a bird (starling)^20^. Two further orbiviruses have been identified from bats (*Myotis rickettsi* and *Rhinolphus ferrumequinum*) in China (Acc. No. KX343070.1 and KX161703.1). All these viruses, Sathuvachari virus, Sathuvachari-like virus discovered in our study and both orbiviruses from China, are closely related to Bluetongue virus 2 and 3 (figure 3). We named the Sathuvachari-like virus Irlbach bat orbivirus (IboV) after the origin of the corresponding bats. The bat rotavirus identified in this study was detected in pool 5 which comprises tissues from *Pipistrellus pipistrellus* bats (Supplementary Table S7). Phylogenetic reconstruction allocates the bat rotavirus sequence into a distinct but related clade to rotaviruses type A (Id 58% nt; 57% aa) (Figure 4). Numerous rotaviruses have been identified in bats but only one other rotavirus has been identified in European bats^5^. The bat rotavirus from France is similarly related to rotaviruses of group A. The zoonotic potential of these bat rotaviruses related to group A has yet to be determined. We named the rotavirus A-like virus Hannover bat rotavirus (HbrV) after the origin of the corresponding bats. A sequence related to other members of the order *Mononegavirales* was identified in intestinal samples of *Nyctalus noctula* bats in pool 2 (Supplementary Table S4). It was described that these bats have parasites in their intestines^21^; it is possible that the sequence originated from a tapeworm. On amino acid level the novel sequence shared highest similarity to Wenzhou tapeworm 1 virus (Id 35% aa) and Midway virus^22, 23^. Phylogenetically the strain was grouped distinct from Midway virus and was more closely related to Borna disease virus and rabies virus. The similarities on nt level are quite low and the tree has to be interpreted with caution (Supplementary Figure S2). However, these findings are interesting as bats are often discussed as potential reservoir host of many viruses of the order *Mononegavirales* (i.e. Ebola virus, Marburg virus, Nipah virus, Hendra virus and lyssaviruses). We named the Wenzhou tapeworm 1-like virus Berlin bat mononegavirus (BbmV) after the origin of the corresponding bats.

**Supplementary tables:**

Supplementary Table S1 Results virus isolation via different cell culture systems

| **Cell line** | **Organism/morphology** | **#/novel/size*** | **Reference cell line** | **Reference novel isolates** |
| --- | --- | --- | --- | --- |
| C6/36 | *Aedes albopictus* (larva, whole) | 0/0/60 | ATCC® CRL-1660^TM^ | - |
| Vero B4 | *Cercopithecus aethiops*  (kidney) | 2/2/60 | DSMZ-ACC33 | T3/Bat/Germany/342/08  (24) |
| Vero E6 | *Cercopithecus aethiops*  (kidney) | 2/2/270 | ATCC® CRL-1586^TM^ | Bat Adenovirus 2  (25, 26) |
| R05T | *Rousettus aegyptiacus*  (fetus head) | 0/0/120 | (27) | - |
| R06T | *Rousettus aegyptiacus*  (fetus head) | 0/0/100 | (27) | - |
| PaKi | *Pteropus alecto*  (kidney) | 0/0/120 | (28) | - |

#/novel/size: #, number of positive samples; novel, number of novel viruses obtained; size, bat sample size

Supplementary Table S2: Results from HiSeq sequencing per pool


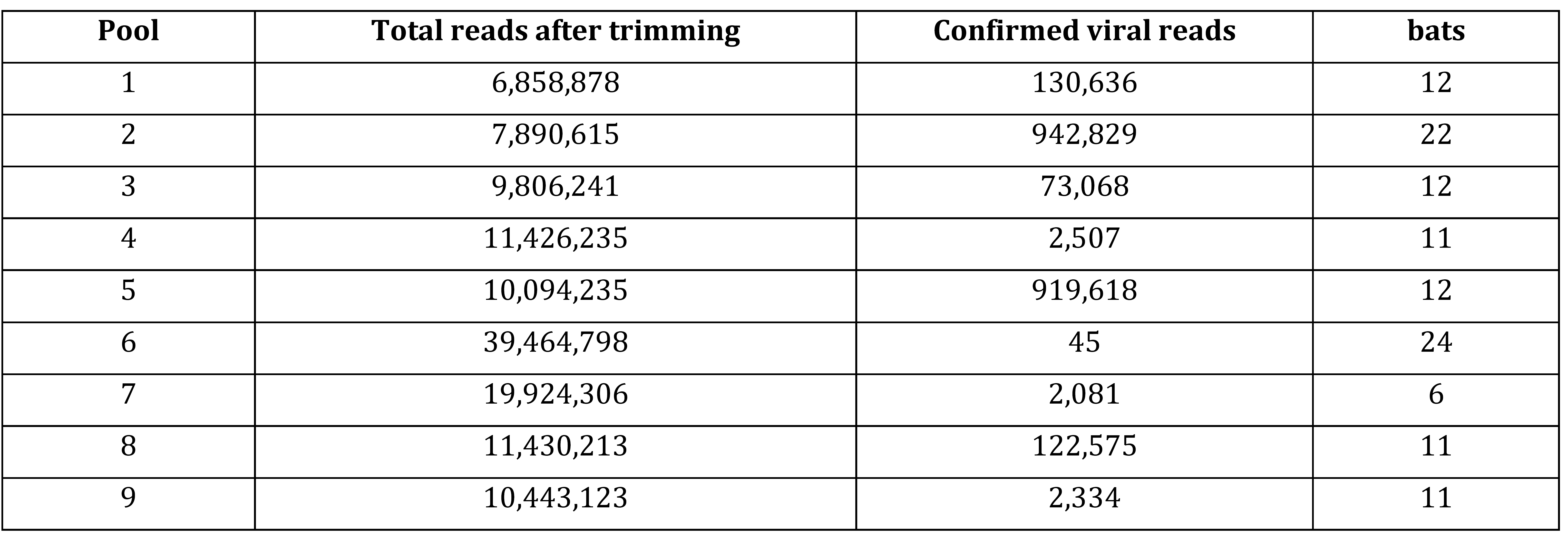


Supplementary Table S3 Results Pool 1


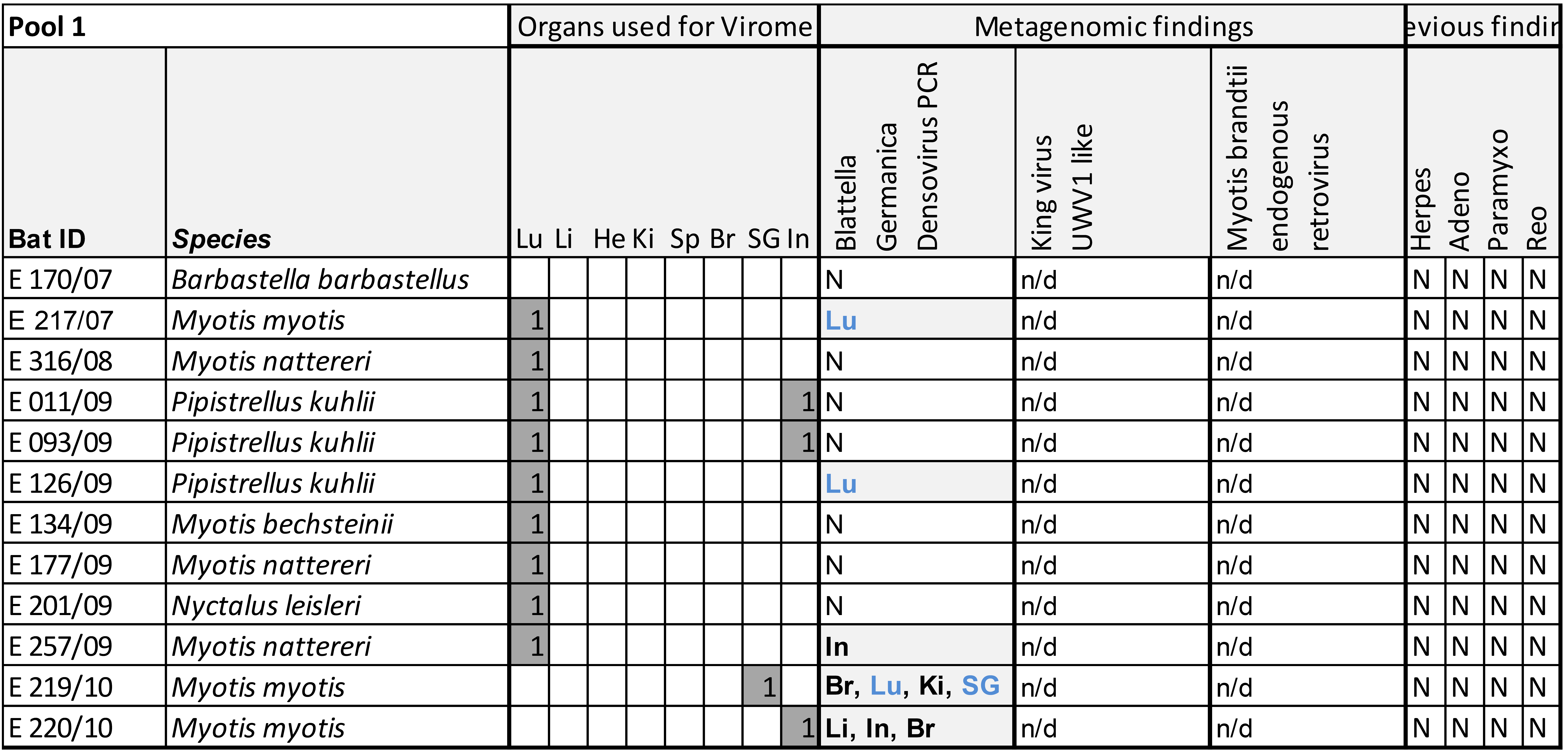


ID, identity; Lu, lungs; Li, liver; He, heart; Ki, kidney; Sp, spleen; Br, brain; SG, salivary glands; In, Intestine; N, not detected; n/d, not determined. Organs indicated in blue: Sanger sequencing performed for this organ and novel virus sequence confirmed.

Supplementary Table S4 Results Pool 2


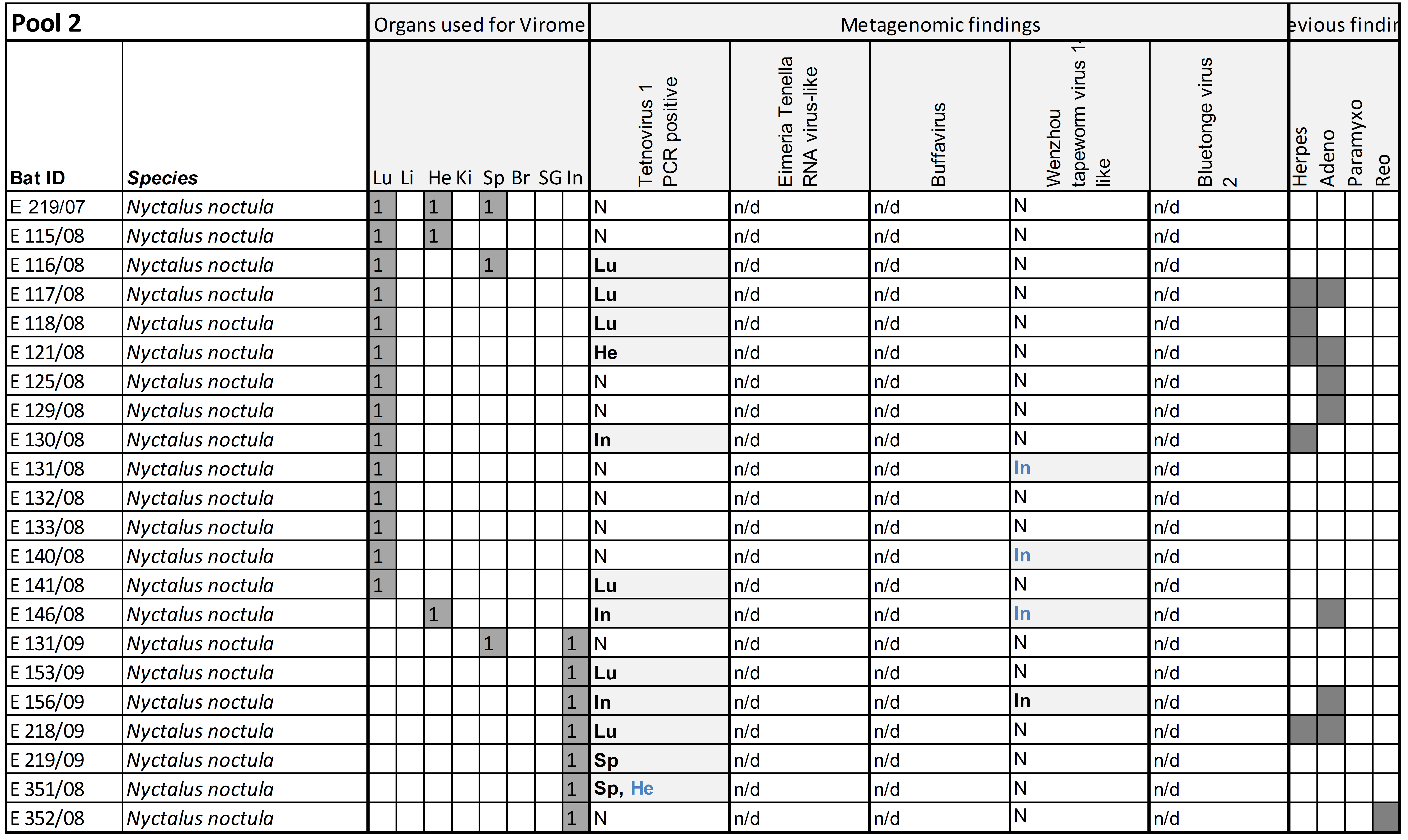


ID, identity; Lu, lungs; Li, liver; He, heart; Ki, kidney; Sp, spleen; Br, brain; SG, salivary glands; In, Intestine; N, not detected; n/d, not determined. Organs indicated in blue: Sanger sequencing performed for this organ and novel virus sequence confirmed.

Supplementary Table S5 Results Pool 3


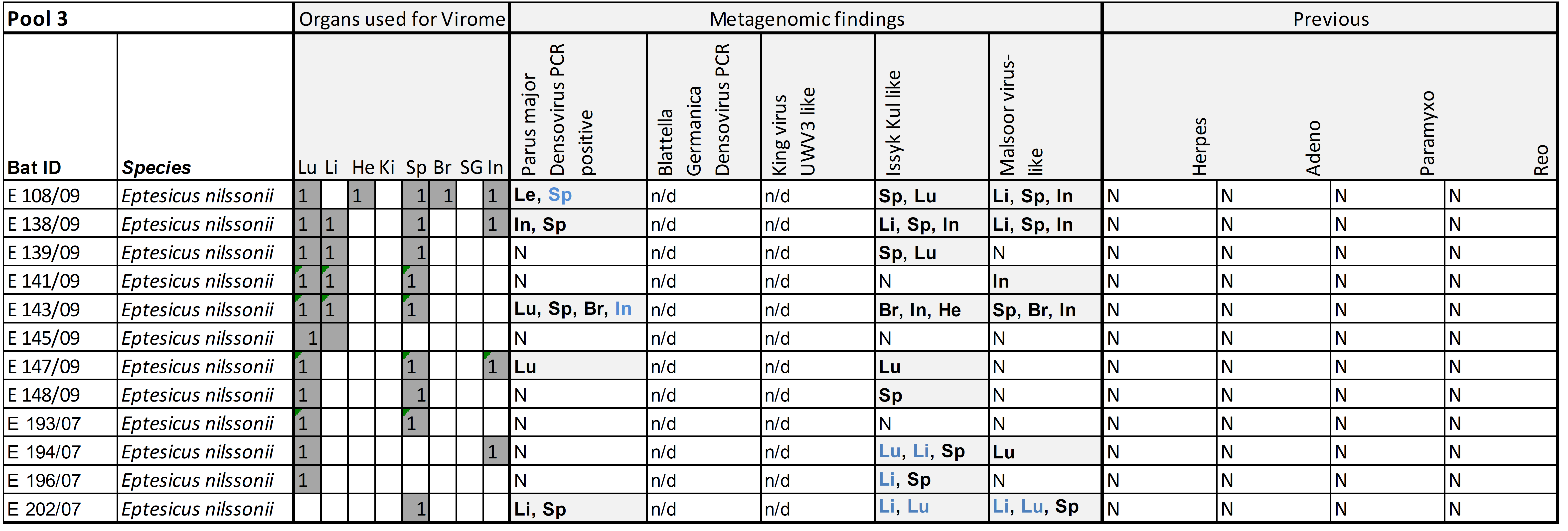


ID, identity; Lu, lungs; Li, liver; He, heart; Ki, kidney; Sp, spleen; Br, brain; SG, salivary glands; In, Intestine; N, not detected; n/d, not determined. Organs indicated in blue: Sanger sequencing performed for this organ and novel virus sequence confirmed.

Supplementary Table S6 Results Pool 4


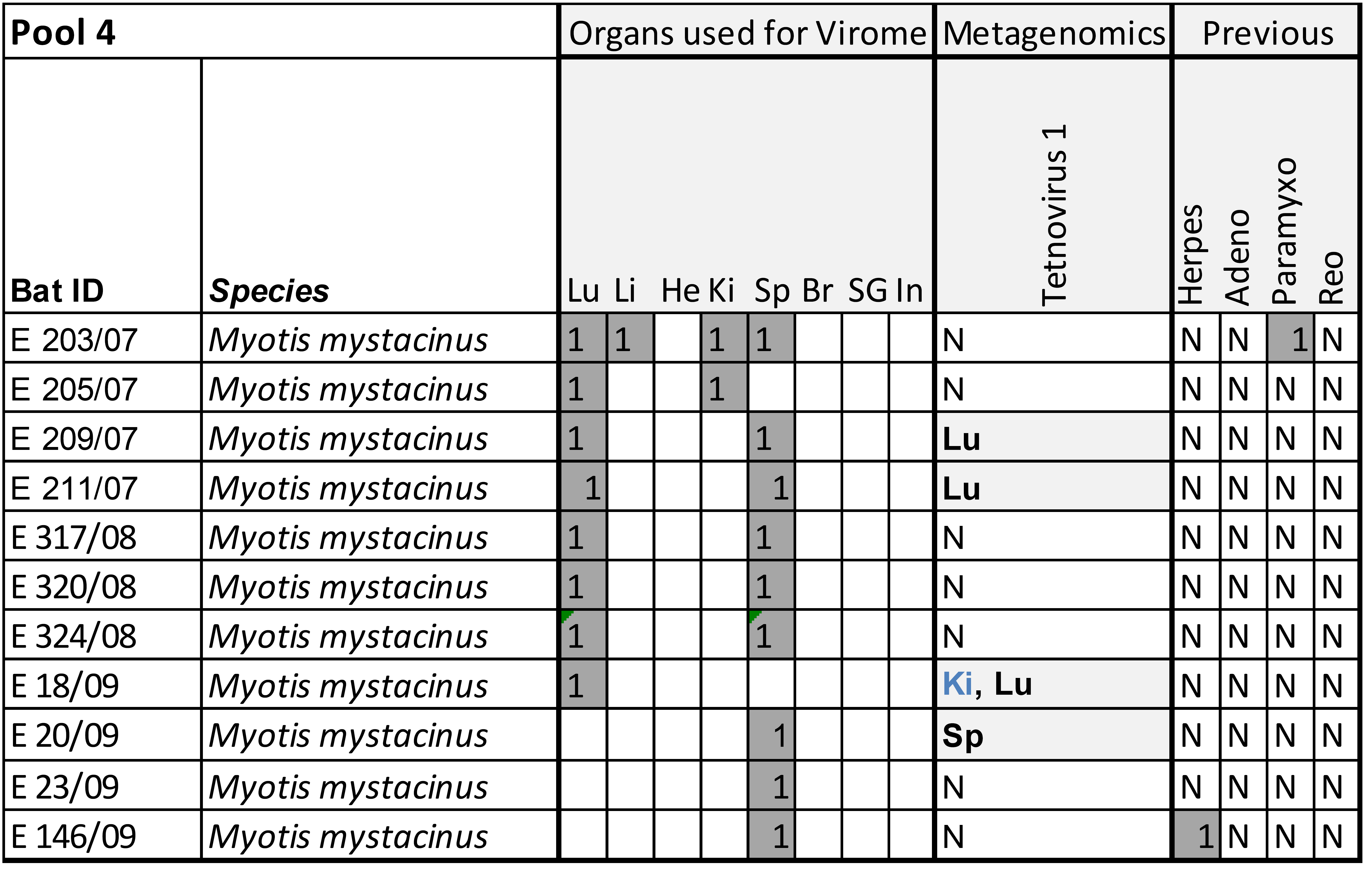


ID, identity; Lu, lungs; Li, liver; He, heart; Ki, kidney; Sp, spleen; Br, brain; SG, salivary glands; In, Intestine; N, not detected; n/d, not determined. Organs indicated in blue: Sanger sequencing performed for this organ and novel virus sequence confirmed.

Supplementary Table S7 Results Pool 5


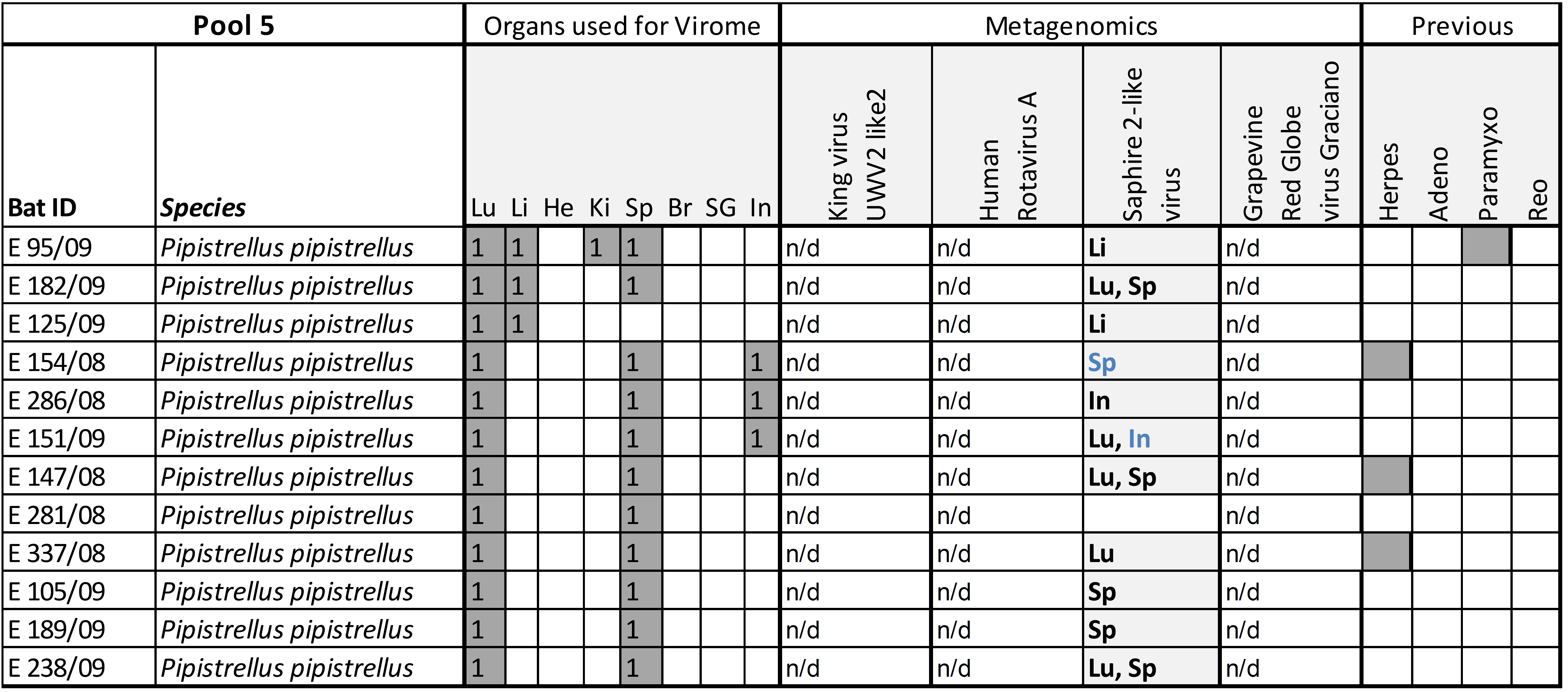


ID, identity; Lu, lungs; Li, liver; He, heart; Ki, kidney; Sp, spleen; Br, brain; SG, salivary glands; In, Intestine; N, not detected; n/d, not determined. Organs indicated in blue: Sanger sequencing performed for this organ and novel virus sequence confirmed.

Supplementary Table S8 Results Pool 6


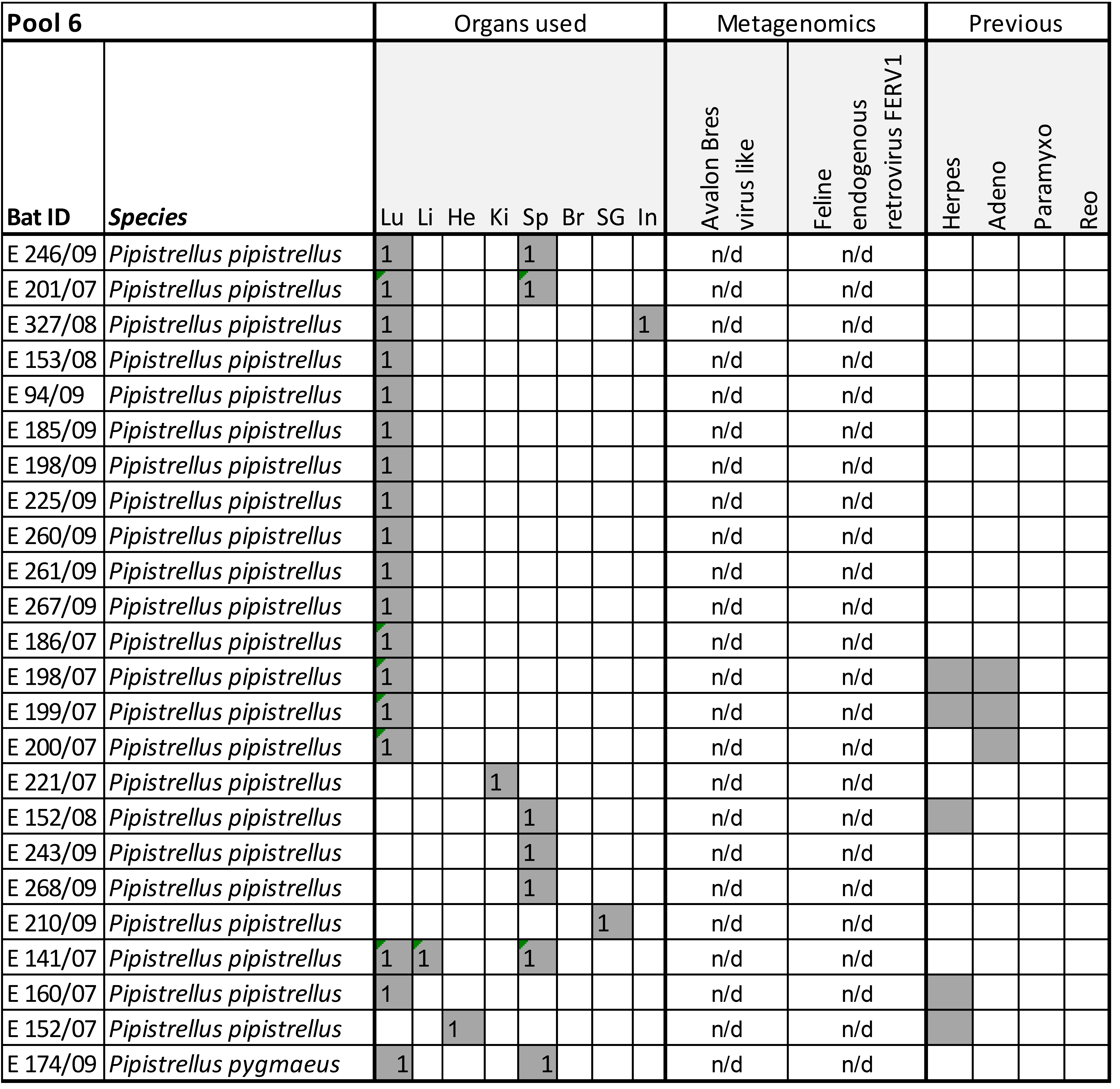


ID, identity; Lu, lungs; Li, liver; He, heart; Ki, kidney; Sp, spleen; Br, brain; SG, salivary glands; In, Intestine; N, not detected; n/d, not determined. Organs indicated in blue: Sanger sequencing performed for this organ and novel virus sequence confirmed.

Supplementary Table S9 Results Pool 7


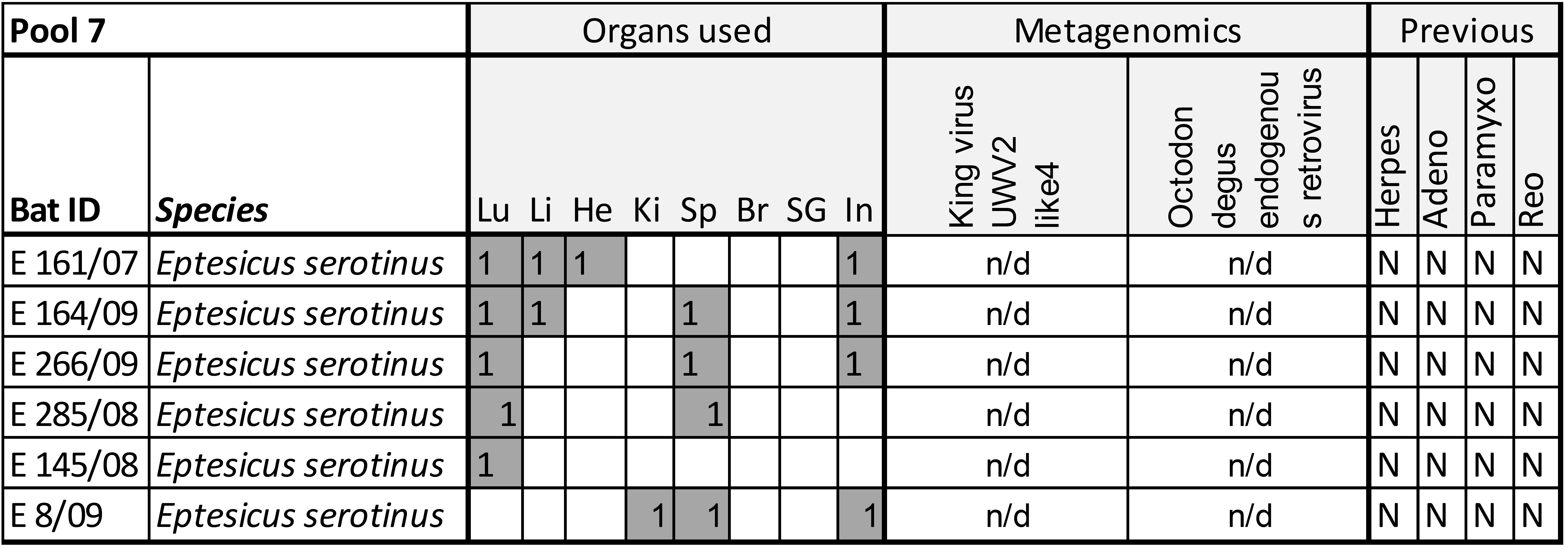


ID, identity; Lu, lungs; Li, liver; He, heart; Ki, kidney; Sp, spleen; Br, brain; SG, salivary glands; In, Intestine; N, not detected; n/d, not determined. Organs indicated in blue: Sanger sequencing performed for this organ and novel virus sequence confirmed.

Supplementary Table S10 Results Pool 8


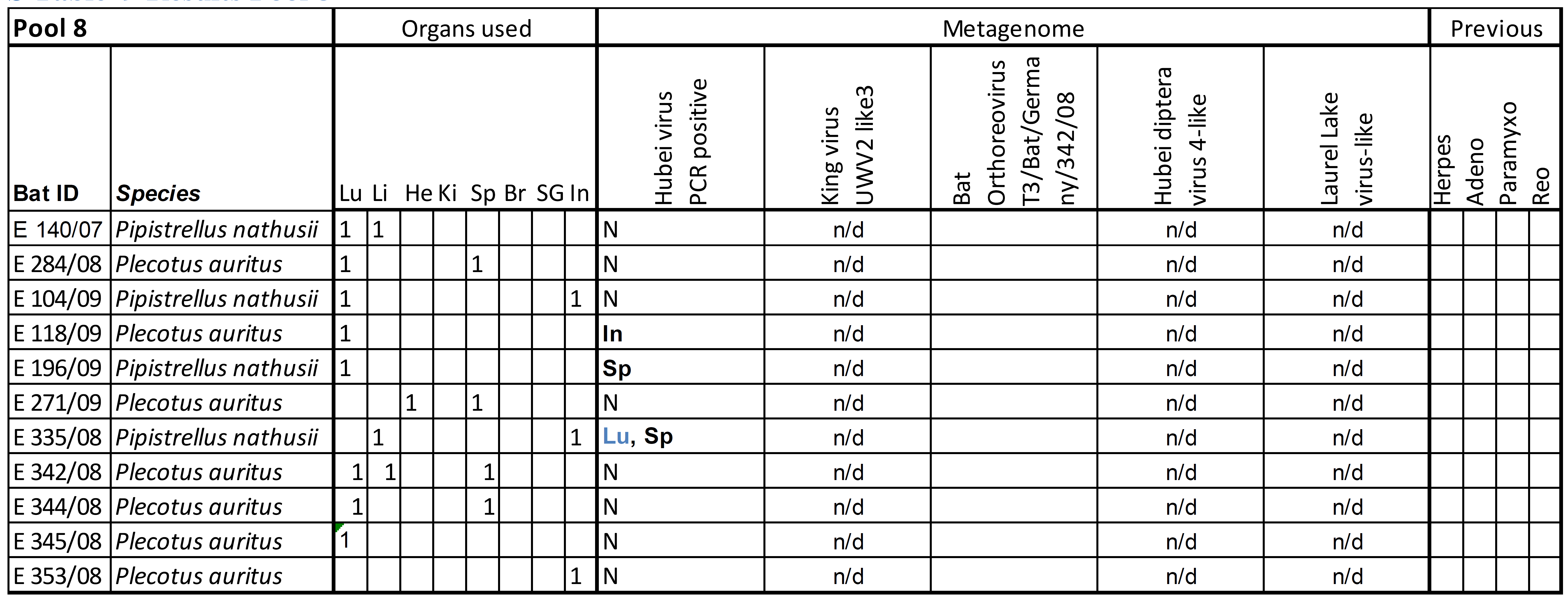


ID, identity; Lu, lungs; Li, liver; He, heart; Ki, kidney; Sp, spleen; Br, brain; SG, salivary glands; In, Intestine; N, not detected; n/d, not determined. Organs indicated in blue: Sanger sequencing performed for this organ and novel virus sequence confirmed.

Supplementary Table S11 Results Pool 9


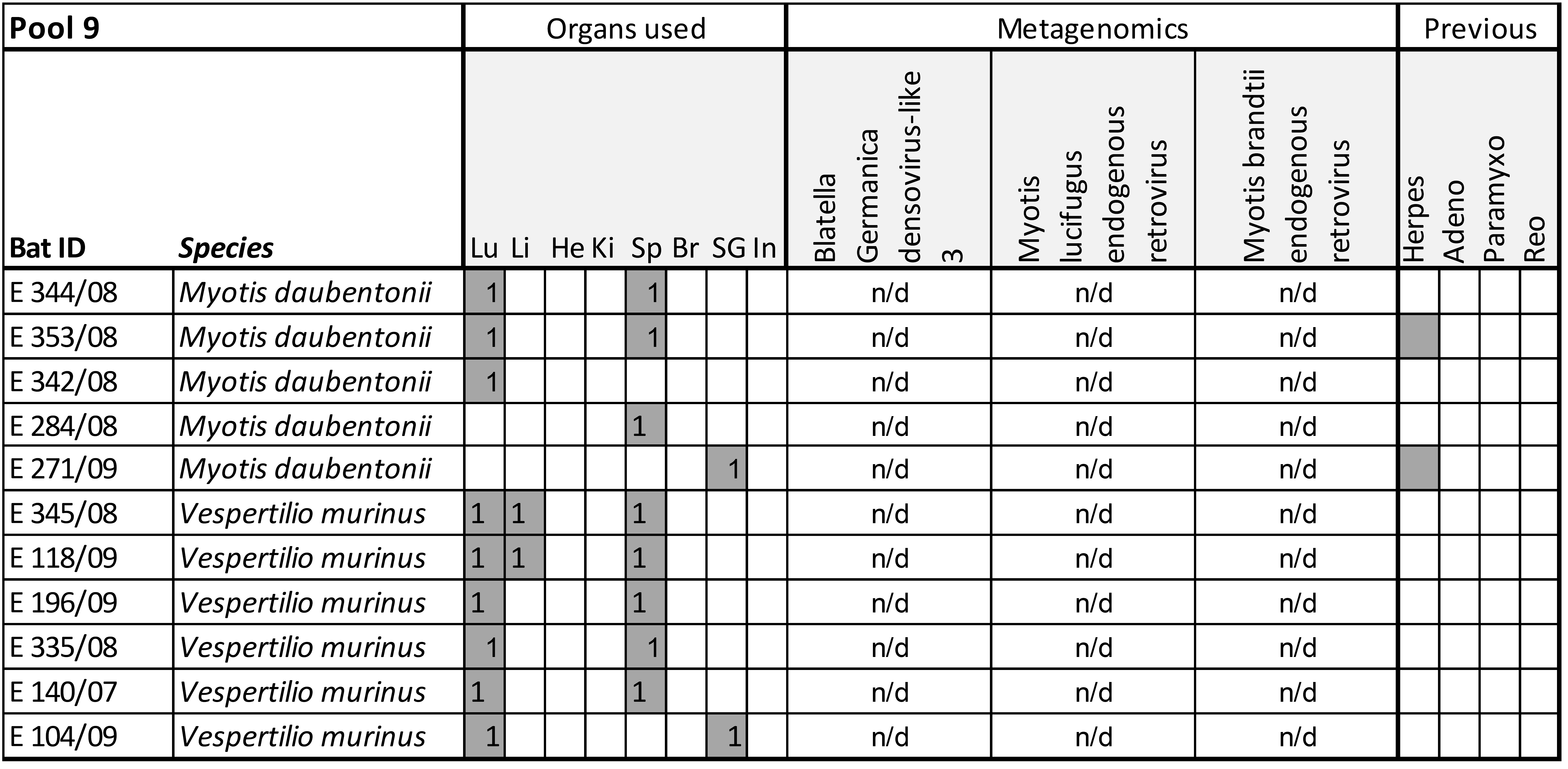


ID, identity; Lu, lungs; Li, liver; He, heart; Ki, kidney; Sp, spleen; Br, brain; SG, salivary glands; In, Intestine; N, not detected; n/d, not determined. Organs indicated in blue: Sanger sequencing performed for this organ and novel virus sequence confirmed.

**Supplementary Figures**

Supplementary Figure S1 Bat CoV tree

Supplementary Figure S2 Phylogenetic reconstruction *Mononegavirales* 665 nt


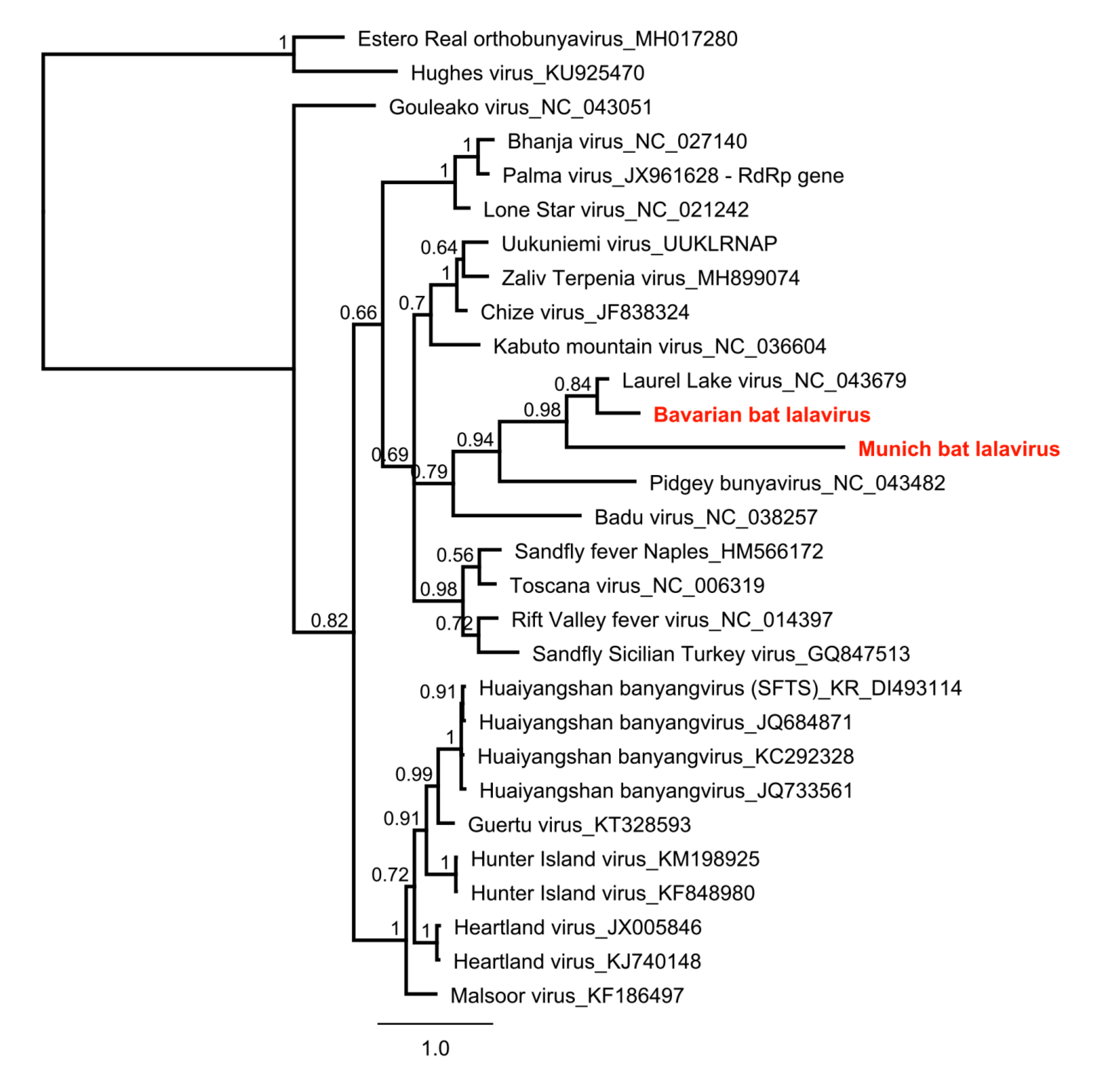


Supplementary Figure S3 Phylogenetic reconstruction 130 nt nucleoprotein (M segment) Phenuiviruses

**Literature Supplement**

1. Huson, D.H., Auch, A.F., Qi, J. & Schuster, S.C. MEGAN analysis of metagenomic data. *Genome Res.* **17,** 377-386 (2007).
2. Buchfink, B., Xie, C. & Huson, D.H. Fast and sensitive protein alignment using DIAMOND. *Nat. Methods* **12,** 59-60 (2015).
3. Rosseel, T., Pardon, B., De Clercq, K., Ozhelvaci, O. & Van Borm, S. False-positive results in metagenomic virus discovery: a strong case for follow-up diagnosis. *Transbound Emerg. Dis.* **61,** 293-299 (2014).
4. Blitvich, B. *et al.* Bunyavirus taxonomy: limitations and misconceptions associated with the current ICTV criteria used for species demarcation. *Am. J. Trop. Med. Hyg.* **99,** 11-16 (2018).
5. Dacheux, L. *et al.* A preliminary study of viral metagenomics of French bat species in contact with humans: identification of new mammalian viruses. *PLoS One* **9,** e87194 (2014).
6. Walker, P.J., Widen, S.G., Wood, T.G., Guzman, H., Tesh, R.B. & Vasilakis, N. A global genomic characterization of nairoviruses identifies nine discrete genogroups with distinctive structural characteristics and host-vector associations. *Am. J. Trop. Med. Hyg.* **94,** 1107-1122 (2016).
7. Salmier, A. *et al.* Virome analysis of two sympatric bat species (Desmodus rotundus and Molossus molossus) in French Guiana. *PLoS One* **12,** e0186943 (2017).
8. Lvov, D.K. *et al.* "Issyk-Kul" virus, a new arbovirus isolated from bats and Argas (Carios) vespertilionis (Latr., 1802) in the Kirghiz S.S.R. Brief report. *Arch. Gesamte Virusforsch*. **42,** 207-209 (1973).
9. Atkinson, B., Marston, D.A., Ellis, R.J., Fooks, A.R. & Hewson, R. Complete genomic sequence of Issyk-Kul virus. *Genome Announc.* **3,** e00662-15 (2015).
10. L'Vov, D.K., Kostiukov, M.A., Daniiarov, O.A., Tukhtaev, T.M. & Sherikov, B.K. Outbreak of arbovirus infection in the Tadzhik SSR due to the Issyk-Kul virus (Issyk-Kul fever). *Vopr. Virusol.* **29,** 89-92 (1984).
11. Brinkmann A. *et al.* First detection of bat-borne Issyk-Kul virus in Europe. *Sci. Rep.* **10,** 22384 (2020).
12. Mahy, B.W.J., *The Dictionary of Virology*, 4th Edition. (Academic Press, 2009).
13. Muller, M.A. *et al.* Evidence for widespread infection of African bats with Crimean-Congo hemorrhagic fever-like viruses. *Sci. Rep*. **6,** 26637 (2016).
14. Mourya, D.T. *et al.* Malsoor virus, a novel bat phlebovirus, is closely related to severe fever with thrombocytopenia syndrome virus and heartland virus. *J. Virol.* **88,** 3605-3609 (2014).
15. Baggieri, M., Marchi, A., Bucci, P., Nicoletti, L. & Magurano, F. Genetic variability of the S segment of Toscana virus. *Virus Res.* **200**, 35-44 (2015).
16. Sall, A.A. et al. Genetic reassortment of Rift Valley fever virus in nature. *J. Virol.* **73,** 8196-8200 (1999).
17. Kohl, C. *et al.* Zwiesel bat banyangvirus, a potentially zoonotic Huaiyangshan banyangvirus (formerly known as SFTS)-like banyangvirus in Northern bats from Germany. *Sci. Rep*. **10,** 1370 (2020).
18. Tokarz, R. *et al.* Identification of novel viruses in Amblyomma americanum, Dermacentor variabilis, and Ixodes scapularis ticks. *mSphere* **3,** e00614-17. <https://doi.org/10.1128/mSphere.00614-17> (2017).
19. Day, J.M. The diversity of the orthoreoviruses: molecular taxonomy and phylogentic divides. *Infect. Genet. Evol.* **9,** 390–400 (2009).
20. Kapoor, A., Tesh, R.B., Duraisamy, R., Popov, V.L., Travassos da Rosa, A.P. & Lipkin, W.I. A novel mosquito-borne Orbivirus species found in South-east Asia. *J. Gen. Virol.* **94,** 1051-1057 (2013).
21. Muhldorfer, K. *et al.* Diseases and causes of death in European bats: dynamics in disease susceptibility and infection rates. *PLoS ONE* **6,** e29773 (2011).
22. Muhldorfer, K., Wibbelt, G., Haensel, J., Riehm, J. & Speck, S. Yersinia species isolated from bats, Germany. *Emerg. Infect. Dis.* **16,** 578-580 (2010).
23. Muhldorfer, K., Speck, S. & Wibbelt, G. Proposal of Vespertiliibacter pulmonis gen. nov., sp. nov. and two genomospecies as new members of the family Pasteurellaceae isolated from E Kohl, C. *et al.* Isolation and characterization of three mammalian orthoreoviruses from European bats. *PLoS One* **7,** e43106 (2012).
24. Kohl, C. *et al.* Genome analysis of bat adenovirus 2: indications of interspecies transmission. *J. Virol.* **86,** 1888-1892 (2012).
25. Sonntag, M., Muhldorfer, K., Speck, S., Wibbelt, G. & Kurth, A. New adenovirus in bats, Germany. *Emerg. Infect. Dis.* **15,** 2052-2055 (2009).
26. Jordan, I., Horn, D., Oehmke, S., Leendertz, FH. and Sandig, V. Cell lines from the Egyptian fruit bat are permissive for modified vaccinia Ankara. *Virus Res* **145**: 54–62 (2009).
27. Crameri G., Todd S., Grimley S., McEachern J.A., Marsh G.A., Smith C., Tachedjian M., De Jong C., Virtue E.R., Yu M., Bulach D., Liu J.P., Michalski W.P., Middleton D., Field H.E. and Wang L.F. Establishment, immortalisation and characterisation of pteropid bat cell lines. *PLoS One*. **11**;4(12):e8266 (2009).
28. uropean bats. *Int. J. Syst. Evol. Microbiol.* **64,** 2424-2430 (2014).
